# Cornelia de Lange syndrome-associated mutations cause a DNA damage signalling and repair defect

**DOI:** 10.1038/s41467-021-23500-6

**Published:** 2021-05-25

**Authors:** Gabrielle Olley, Madapura M. Pradeepa, Graeme R. Grimes, Sandra Piquet, Sophie E. Polo, David R. FitzPatrick, Wendy A. Bickmore, Charlene Boumendil

**Affiliations:** 1 MRC Human Genetics Unit, Institute of Genetics and Cancer, University of Edinburgh, Crewe Road, Edinburgh, UK; 2 Blizard institute, Barts and The London School of Medicine and Dentistry, Queen Mary University of London, London, UK; 3 Epigenetics and Cell Fate Centre, UMR7216 CNRS, Université de Paris, Paris, France; 4 Université de Paris, CNRS, Institut Jacques Monod, Paris, France

**Keywords:** Gene regulation, Genomic instability

## Abstract

Cornelia de Lange syndrome is a multisystem developmental disorder typically caused by mutations in the gene encoding the cohesin loader NIPBL. The associated phenotype is generally assumed to be the consequence of aberrant transcriptional regulation. Recently, we identified a missense mutation in BRD4 associated with a Cornelia de Lange-like syndrome that reduces BRD4 binding to acetylated histones. Here we show that, although this mutation reduces BRD4-occupancy at enhancers it does not affect transcription of the pluripotency network in mouse embryonic stem cells. Rather, it delays the cell cycle, increases DNA damage signalling, and perturbs regulation of DNA repair in mutant cells. This uncovers a role for BRD4 in DNA repair pathway choice. Furthermore, we find evidence of a similar increase in DNA damage signalling in cells derived from NIPBL-deficient individuals, suggesting that defective DNA damage signalling and repair is also a feature of typical Cornelia de Lange syndrome.

## Abstract (web summary)

Cornelia de Lange syndrome is a developmental disorder typically caused by mutations in the gene encoding the cohesin loader NIPBL. The authors, here, by analysing previously identified mutations in BRD4 associated with the disease, reveal that a BRD4 mutation affects DNA damage signalling, and perturbs regulation of DNA repair in mutant cells.

## Introduction

Cornelia de Lange syndrome (CdLS) is a clinically distinctive neurodevelopmental disorder (OMIM:122470). Disease severity varies greatly and patients can suffer from a range of symptoms including: a characteristic facial appearance, upper limb abnormalities, intellectual disability and delayed growth^1^. CdLS is described as a ‘cohesinopathy’^1^—most cases can be attributed to heterozygous loss of function mutation in *NIPBL* encoding a protein involved in loading of the cohesin complex onto chromatin^2^. Mutation in genes encoding cohesin complex proteins SMC1, SMC3 and RAD21, or HDAC8 (SMC3 deacetylase), have also been identified in CdLS-like probands^2^. However cells from CdLS patients have no obvious defects in sister chromatid cohesion^3^, and individuals with mutations in *SMC1, SMC3* and *RAD21* are often considered ‘atypical’ in terms of facial appearance and growth, and are less likely to have limb defects than those with *NIPBL* mutations^4^.

Dysregulated gene expression has been proposed to be the main mechanism underlying CDLS^5,6^. Mutations in genes encoding chromatin regulators unrelated to cohesin, such as ANKRD11, KMT2A, AFF4 and the bromodomain and extra-terminal domain (BET) protein BRD4, have been reported to cause CdLS-like phenotypes^1^ suggesting that chromatin dysregulation may play a role in CdLS as well. Additionally, increased sensitivity to DNA damage has been reported in CdLS patient cells^7^, but the mechanism underlying this defect is unknown and its participation in the disease aetiology remains unclear.

Recently, we described de novo deletion and missense mutations in *BRD4* associated with a clinical phenotype overlapping CdLS^8^. BRD4 binds acetylated lysine residues in histones H3 and H4 through its two N-terminal bromodomain domains (BD). BRD4 localises to promoters and enhancers of active genes and is particularly enriched at super enhancers (SEs)^9,10^. BRD4 is a key regulator of transcription; through its C-terminal domain it recruits positive transcription elongation factor (P-TEFb) and the Mediator complex to promoters and enhancers, whilst its extra-terminal domain confers transcriptional activation through the recruitment of CHD4, JMJD6 and NSD3^11,12^.

**Fig. 1:**
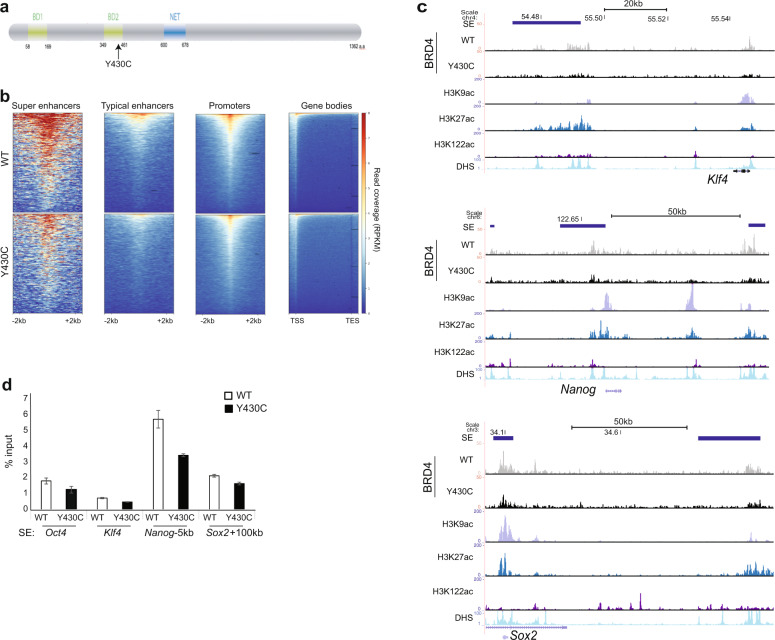
Decreased binding of BRD4 at CREs in Y430C mESCs. **a** Cartoon of BRD4 showing location of the Y430C mutation in the second bromodomain (BD2). **b** Heatmaps show enrichment of wild-type (WT) and Y430C BRD4 ChIP over super enhancers (SE), typical enhancers, promoters and gene bodies. **c** UCSC genome browser screenshot showing reads per 10 million over the Klf4, extended Nanog and Sox2 loci for BRD4 ChIP-seq in WT and Y430C mESCs. Extent of SEs are shown in blue. Below are shown previously published ChIP-seq data for H3K27ac (ENCSR000CDE), H3K9ac (ENCSR000CGS), H3K122ac (GSE66023) and DNase I hypersensitivity (DHS). Genome co-ordinates (Mb) are from the mm9 assembly of the mouse genome. Biological replicate from an independent Y430C clone are in Supplementary Fig. 1. **d** ChIP-qPCR measuring concentration of BRD4 ChIP DNA relative to input across the SEs of Oct4, Klf4, Nanog, and Sox2; in WT and Y430C mESCs. Data are represented as mean ± SEM from 3 technical replicates.

The CdLS-associated BRD4 missense mutation is in the second bromodomain (BD2) (NM_058243.2:c.1289A > G, p.(Tyr430Cys), termed here as Y430C (Fig. 1a), and results in decreased binding to acetylated histones^8^. To gain further insights into the mechanisms underlying CdLS, and the role of BRD4, we investigated the phenotype of mouse embryonic stem cells (mESCs) homozygous for the orthologous amino acid substitution in mouse Brd4 (actually p.Tyr431Cys but for simplicity here termed *Brd4*^*Y430C*^). Here we show that the decreased affinity for acetylated lysines results in diminished occupancy of BRD4^Y430C^ at *cis* regulatory elements (CREs) across the genome, including the super-enhancers of pluripotency genes. However, we find no evidence of altered transcription of the pluripotency network in these cells. Instead, we report increased and more persistent DNA damage signalling and cell cycle checkpoint activation in *Brd4*^*Y430C*^ mESCs. We show increased persistent foci of the DNA damage response (DDR) protein 53BP1 upon double-strand break (DSB) induction in Brd4 mutant cells. 53BP1 is a key factor in the regulation of DNA repair pathway choice that inhibits repair by homologous recombination (HR). We also show increased foci of the downstream effectors of 53BP1, Rif1 and the Mad2l2 (Rev7) subunit of the shieldin complex in the mutant cells^13–22^ and decreased recruitment of RAD51, suggesting impaired HR repair. Further, we show that cells from CdLS patients harbouring mutations in *NIPBL* have a similar DDR phenotype, indicating there may be a previously underappreciated role for the DNA damage response in the aetiology of CdLS.

## Results

### Reduced occupancy of Y430C-BRD4 at *cis*-regulatory elements

Our previous work suggested that the Y430C mutation abrogates BRD4 binding to acetylated histones in vitro and in vivo. To determine the genome-wide effect of this reduced affinity we carried out BRD4 ChIP-seq in two independently generated mESCs lines engineered by CRISPR-Cas9 to carry the Y430C mutation on both alleles of *Brd4*. Immunoblotting showed that the Y430C mutant BRD4 protein was present at levels equivalent to that of BRD4 in wild-type cells (Supplementary Fig. S1a) and was efficiently immunopreciptated by anti-Brd4 antibodies (Supplementary Fig. S1b). As expected, BRD4 was enriched over CREs (SEs, typical enhancers and promoters) in both wild-type (WT) and Y430C cells (Fig. 1b, Supplementary Fig. S1c). However, consistent with a lowered affinity for acetyl-lysines, there was a general decrease in BRD4 occupancy in both clones of Y430C cells, most striking at enhancers and super-enhancers (SE) (Fig. 1c, d, Supplementary Fig. S1c–d). In mESCs, BRD4 binding to SEs regulates the transcription of stem cell identity genes^9^. As BRD4^Y430C^ occupancy is decreased at the SEs of a number of stem cell identity genes, this suggests that there might be decreased transcription of these genes in mutant cells.

### Decreased occupancy of mutant BRD4 at CREs does not have major effects on transcription in mESCs

**Fig. 2:**
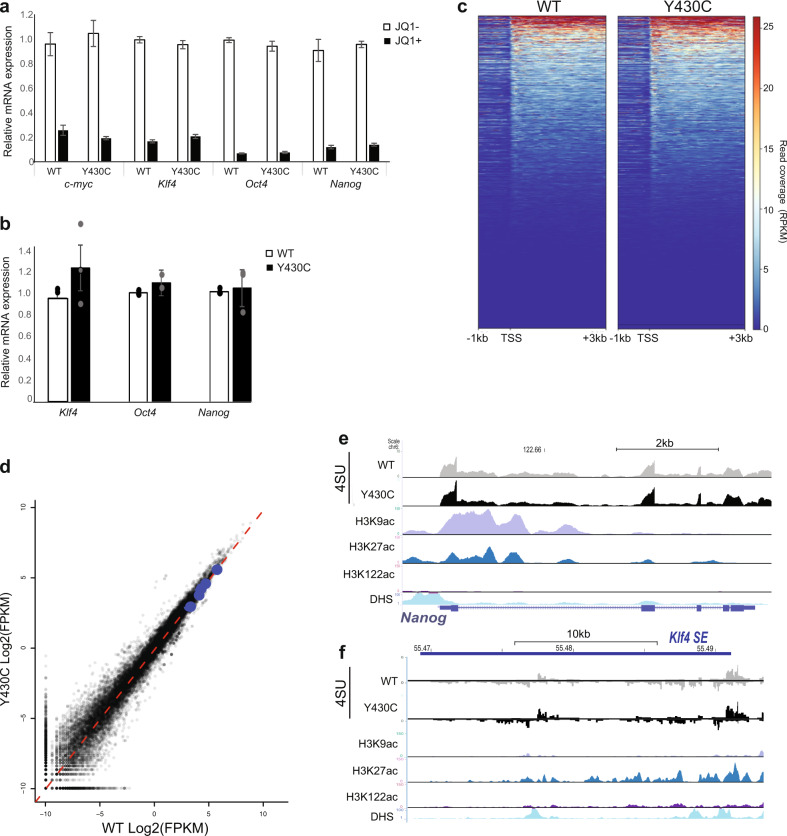
Similar transcription in WT and Y430C mESCs. **a** RT-qPCR measuring mRNA of c-myc, Klf4, Oct4 and Nanog in mESCs after treatment with 300 nM JQ1 + , relative to that in untreated cells (JQ1-). Data are represented as mean ± SEM from 3 technical replicates. **b** RT-qPCR measuring mRNA for Klf4, Oct4 and Nanog in WT and Y430C mESCs. mRNA concentration is shown relative to WT set at 1. Data are represented as mean ± SEM from 3 biological replicates. **c** Heatmaps show enrichment of 4sU-seq data in WT and Y430C cells over transcribed regions (−1kb, TSS and +3 kb) (mm9_refseq). **d** Scatter plot of the 4sU-seq data in WT and Y430C cells, highlighting pluripotency genes in blue (Nanog, Sox2, Klf4, Esrrb, Pou5f1). Red dashed line shows best fitted line. Pearson correlation coefficient = 0.98. **e**, **f** UCSC browser screenshot showing 4SU-seq reads per 10 million over **e** the Nanog locus and **f** the Klf4 super-enhancer in WT and Y430C cells and ChIP-seq tracks for various histone modifications and DNaseI hypersensitivity in WT cells. Genome co-ordinates (Mb) are from the mm9 assembly of the mouse genome. Data from a biological replicate Y430C clone are in Supplementary Fig. 2.

The use of inhibitors that competitively bind the acetyl-lysine binding pockets of BET proteins has shown that loss of BRD4 binding disrupts the expression of target genes, especially genes regulated by SEs^10^. Consistent with this, we observed decreased expression of the SE associated genes *Nanog, Myc, Klf4* and *Oct4* in both WT and Y430C mESCs after treatment with JQ1 (Fig. 2a). However, we did not observe any decrease in levels of *Klf4*, *Nanog* and *Oct4* mRNAs in Y430C compared to WT mESCs by RT-qPCR (Fig. 2b).

To determine whether mRNA stability was masking an effect on transcription per se, we performed 4-thiouridine sequencing (4SU-seq) to assay nascent transcription. Transcription was surprisingly similar between WT and Y430C mESCs (Pearson correlation coefficient = 0.98) (Fig. 2c, d and data from an independent Y430C in Supplementary Fig. 2a). In particular, decreased BRD4 binding at SEs did not lead to transcriptional changes at stem cell identity genes (Fig. 2c–e, replicate in Supplementary Fig. 2b), or of eRNAs at the SEs themselves (Fig. 2f, Supplementary Fig. 2c). Due to normalisation, these experiments could not rule out that transcription is not globally decreased in the mutant ESCs. We therefore performed a spike-in RNA-seq experiment, using RNA from *Drosophila* cells for normalisation. Again, we did not observe any major transcriptional differences between WT and Y430C cells (Supplementary Fig. 2d, e). We conclude that the decreased occupancy of BRD4^Y430C^ at CREs in mESCs is not sufficient to affect the transcription of associated genes.

This result is surprising, given BRD4’s well-documented roles in transcriptional regulation. Therefore, we also analysed gene expression in embryonic fibroblasts (MEFs) that had been derived from either wild-type or *Brd4*^*Y430C*^ homozygous embryos^8^. In contrast to homozygous null Brd4 embryos, which show significant growth retardation at E13.5 of embryonic development, *Brd4*^*Y430C*^ homozygous embryos are reported to be morphologically indistinguishable from wild-type at this stage^8^ suggesting the absence of major developmental gene dysregulation. However, analysis of gene expression shows significant alteration of gene expression between wild-type and mutant MEFS (Supplementary Fig. 3). We consider that this difference in the transcriptional consequences of Brd4^Y430C^ between ESCs and MEFS may reflect the differing dependencies on epigenetic regulators at different stages of development.

### Y430C-BRD4 mESCs have a delayed cell cycle and increased cell cycle checkpoint activation

**Fig. 3:**
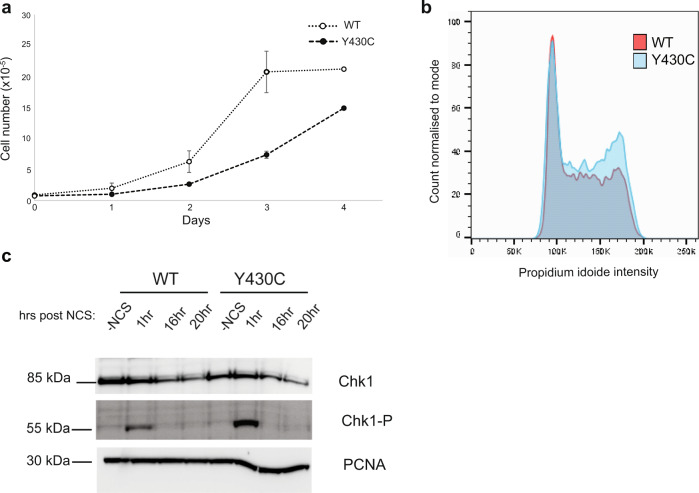
Increased G2/M checkpoint activation in Y430C mESCs. **a** Graph shows average number of WT and Y430C cells per well at 1, 2, 3 and 4 days post seeding. Data are represented as mean ± SEM from 3 technical replicates. **b** Overlaid graphs show WT and Y430C cell cycle profiles, as determined by flow cytometry. Graphs illustrate the cell count, which correlates to propidium iodide intensity. Biological replicate in Supplementary Fig. 4a. **c** Immunoblot using antibodies against Lamin B CHK1 and CHK1-P after treatment of WT and Y430C mESCs with NCS and for various times (hrs) of recovery (source data are provided as a Source Data file). Data from an independent experiment are presented in Supplementary Fig. 4.

We noted that *BRD4*^*Y430C*^ mESCs grew slower and showed an accumulation of cells in G2/M (33.7%), compared to their WT counterparts (27.8%) (Fig. 3a, b, Supplementary Fig. 4a and Supplementary Fig. 9). This observation, together with the recently reported roles for BRD4 in the DDR and DNA repair^23–26^ led us to investigate potential DDR defects in mutant cells.

The DDR allows coordination between DNA repair and cell cycle progression. Recognition of DNA damage by sensor proteins initiates a cascade that results in the phosphorylation and activation of the checkpoint kinases CHK1 and CHK2, delaying or blocking cell cycle progression^27^. CHK1 is the main kinase required for delay at G2/M^27^. To determine whether the altered cell cycle in *BRD4*^*Y430C*^ cells is associated with increased activation of the G2/M checkpoint, we analysed CHK1 phosphorylation (CHK1-P) after treatment with neocarzinostatin (NCS), a radiomimetic drug which induces mainly DSBs. CHK1-P is increased in both WT and Y430C mESCs cell lines 1 h post NCS treatment, which is resolved by 16 h. However, the levels of CHK1-P are higher in *BRD4*^*Y430C*^ mESCs (Fig. 3c, Supplementary Fig. 4b), suggesting an increased checkpoint activation.

These results suggest a defect in DNA repair or signalling caused by BRD4^Y430C^. BRD4 has been shown to be directly involved in DNA repair through the transcriptional regulation of DNA repair proteins^24,25,28^. However, 4SU-seq showed that transcription of genes encoding DNA repair proteins was unaffected in *BRD4*^*Y430C*^ mESCs (Supplementary Fig. 4c, d) and immunoblotting showed that overall levels of 53BP1 and Rad51 were not significantly altered in the mutant cells (Supplementary Fig. 4e).

### Y430C-BRD4 mESCs have increased DDR signalling

Although not being directly recruited to DSBs^29^ (Supplementary Fig. 5a), BRD4 restricts the DDR and depletion of BRD4 isoform B leads to increased DDR signalling^23^. We therefore tested whether BRD4^Y430C^ affects DNA damage signalling. mESCs have constitutively high levels of γH2AX, even in the absence of a DNA damaging stimulus^30^. We therefore used 53BP1 as a marker of DDR. 53BP1 is recruited to DSBs, spreads to form microscopically visible foci and acts as a scaffold for the recruitment of further DSB response proteins, to regulate the choice of DNA repair pathway and to promote cell cycle checkpoint signalling^31^.

**Fig. 4:**
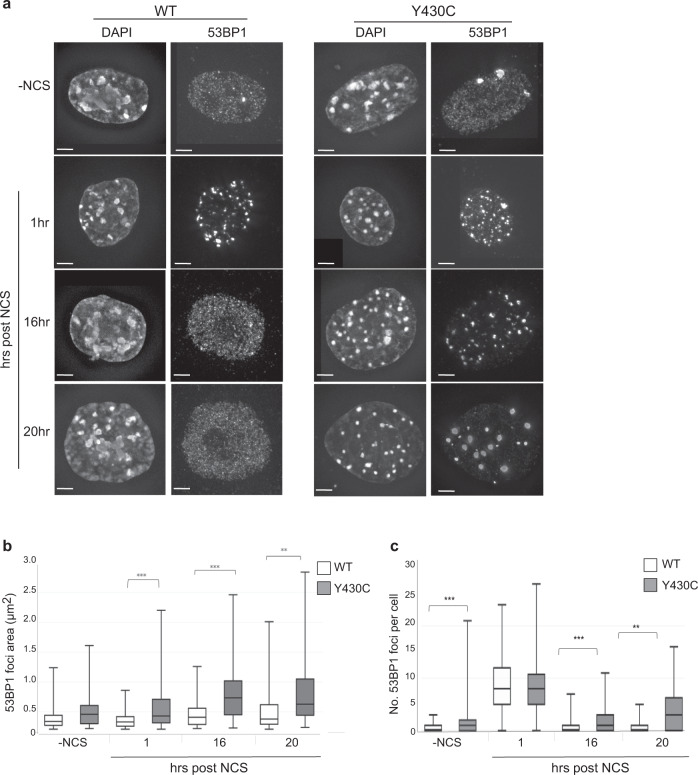
Increased size and number of 53BP1 foci after DSB induction in Y430C mESCs. **a** Immunofluorescence for 53BP1 in the DAPI-stained nuclei of wild-type and Y430C mESCs upon treatment with NCS and after recovery periods up to 20 h. Scale bars: 2 μm. The experiment was repeated 3 times independently with similar results. **b**, **c** Box-plots show area (μm^2^) and number of 53BP1 foci per cell, respectively, in WT and Y430C cells after treatment with NCS in one representative experiment. Horizontal lines within boxes show medians, boxes are inter-quartile ranges and whiskers are range. *P*-values were calculated with Mann–Whitney one-sided *U* test. *<0.05, **<0.01, ***<0.001. 50 cells per condition were examined. Data from three independent experiment are in Supplementary Fig. 6.

Immunofluorescence showed formation of multiple 53BP1 foci, representing DNA damage sites, upon DSB induction (1 h after NCS treatment). These foci are only present at low levels prior to NCS treatment and decrease in number at 16 and 20 h post treatment, as cells repair the damage (Fig. 4a). Supporting the hypothesis that the Y430C mutation impairs the role of BRD4 in DDR restriction, we observed that 53BP1 foci are larger in *BRD4*^*Y430C*^ mESCs than in WT (Fig. 4a, b). In addition, whilst the number of 53BP1 foci in WT cells returns to pre-treatment levels at 16 and 20 h time points, the number of 53BP1 foci in *BRD4*^*Y430C*^ cells remains higher (Fig. 4a, c, Supplementary Fig. 5b, c), suggesting that DNA repair itself could be impaired.

During our analysis of transcription, we found potential evidence for aneuploidy for chromosome 11 in *BRD4*^*Y430C*^ cells. Concerned that the DNA repair defects we observed in *BRD4*^*Y430C*^ cells were caused by aneuploidy rather than the BRD4 mutation, we repeated the 53BP1 staining using WT and *BRD4*^*Y430C*^ mESCs that had been confirmed to have a diploid karyotype. This analysis confirmed that the increased number and size of 53BP1 foci after NCS treatment is specific to cells carrying the *BRD4*^*Y430C*^ mutation, and not an additional chromosome 11 (Supplementary Fig. 6).

Given the accumulation of *BRD4*^*Y430C*^ cells in S/G2 (Fig. 3), one possible origin of 53BP1 foci is the formation of Oct1/PTF/transcription (OPT) domains arising as a consequence of problems encountered in S-phase and propagated through mitosis into the subsequent G1^32,33^. Indeed inhibition of BRD4 by BET inhibitors has been reported to induce replication stress^34^. However, immunofluorescence showed that 53BP1 foci present in *BRD4*^*Y430C*^ cells, either with or without NCS treatment, do not co-localise with OPT domains marked by Oct1 (Supplementary Fig. 7).

### Defective DSB repair in Y430C-BRD4 cells

For the most part, DSBs are repaired by either non-homologous end-joining (NHEJ) or HR^35^. Use of the appropriate pathway is important for faithful repair and is determined by antagonistic recruitment of 53BP1 and BRCA1^31^. 53BP1 inhibits DSB end resection, the initial step of HR, thereby promoting NHEJ and inhibiting HR. Downstream effectors of 53BP1 in the regulation of resection include RIF1^19–22^ and the recently identified shieldin complex (SHLD1, SHLD2, SHLD3 and MAD2L2)^13–18^. If timely repair does not occur by NHEJ, BRCA1 promotes the release of RIF1, leading to end-resection and HR.

**Fig. 5:**
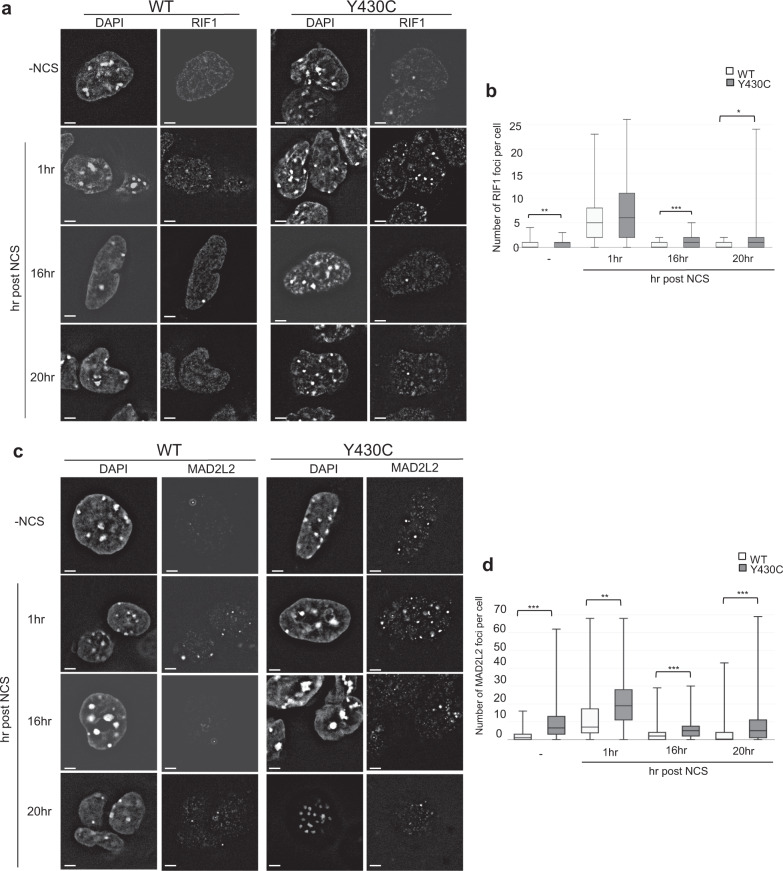
Increased RIF1 and MAD2L2 foci after DSB induction in Y430C mESCs. **a** Representative images of wild-type and Y430C mESCs upon RIF1 immunofluorescence and DAPI staining after treatment with NCS. Scale bars: 2 μm. The experiment was repeated three times with similar results. **b** Box-plot shows number of RIF1 foci per cell, respectively, in WT and Y430C cells after treatment with NCS in one representative experiment. Horizontal lines within boxes show medians, boxes are inter-quartile ranges and whiskers are range. *P*-values were calculated with Mann–Whitney one-sided *U* test. *<0.05, **<0.01, ***<0.001. 50 cells were examined per condition. **c** Representative images of wild-type and Y430C mESCs upon MAD2L2 immunofluorescence and DAPI staining after treatment with NCS. Scale bars: 2 μm. The experiment was repeated three times with similar results. **d** Box-plot shows number of MAD2L2 foci per cell, respectively, in WT and Y430C cells after treatment with NCS in one representative experiment. Horizontal lines within boxes show medians, boxes are inter-quartile ranges and whiskers are range. *P*-values were calculated with Mann–Whitney one-sided *U* test. *<0.05, **<0.01, ***<0.001. 50 cells were examined per condition.

**Fig. 6:**
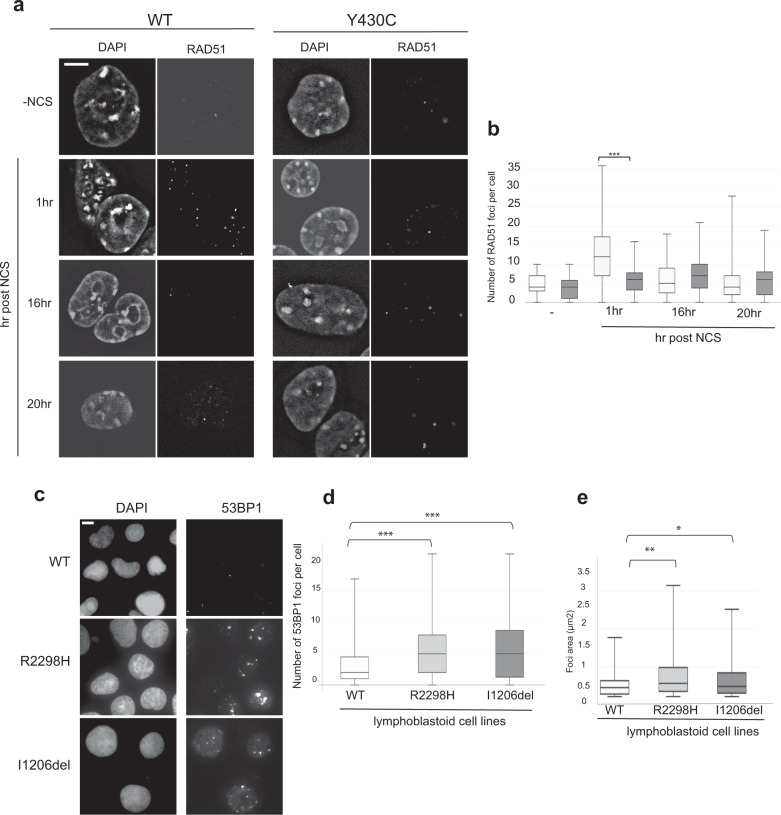
Evidence for DNA repair defects in CdLS. Representative images of wild-type and Y430C mESCs upon RAD51 immunofluorescence and DAPI staining after treatment with NCS. Scale bar = 5 μm. The experiment was repeated three times with similar results. **b** Box-plot shows number of RAD51 foci per cell, respectively, in WT and Y430C cells after treatment with NCS in one representative experiment. Horizontal lines within boxes show medians, boxes are inter-quartile ranges and whiskers are range. *P*-values were calculated with Mann–Whitney one-sided *U* test. *<0.05, **<0.01, ***<0.001. 50 cells were examined per condition. **c** Representative images of wild-type, R2298 and I1206del LCLs upon 53BP1 and DAPI immunofluorescence. Scale bar = 5 μm. The experiment was repeated three times with similar results. **d**, **e** Box-plots show number of 53BP1 foci per cell and area of 53BP1 foci (μm^2^), respectively, in WT, R2298H and I1206del LCLs in one representative experiment. Horizontal lines within boxes show medians, boxes are inter-quartile ranges and whiskers are range. *P*-values were calculated with Mann–Whitney one-sided *U* test. *<0.05, **<0.01, ***<0.001. 50 cells were examined per condition.

As *BRD4*^*Y430C*^ mESCs show increased numbers and size of 53BP1 foci compared to WT cells, we reasoned that there may also be increased recruitment of the downstream effectors of 53BP1 such as RIF1 and MAD2L2. Indeed, we observed an increased number of RIF1 (Fig. 5a, b, Supplementary Fig. 5d, e) and MAD2L2 (Fig. 5c, d, Supplementary Fig. 5f, h) foci in *BRD4*^*Y430C*^ compared to WT cells at all time-points, similar to 53BP1. Conversely, we observed a significant decrease in the number of foci of RAD51, a protein necessary for HR repair, in mutant cells at 1 h post NCS (Fig. 6a, b, Supplementary Fig. 5h, i), suggesting a repression of HR. Given our cell cycle analysis (Fig. 3b) this cannot be simply explained by fewer cells in S/G2. Given the role of the shieldin complex in protecting DSB end-resection, we propose that the Y430C BRD4 mutation leads to an altered balance between NHEJ and HR, consistent with the synthetic lethality observed between BRD4 and PARP inhibitors^25,28^.

### Increased number and size of 53BP1 foci in NIPBL mutant lymphoblastoid cell lines

To see if the DDR defect that we have observed in the presence of the BRD4^Y430C^ would also be apparent in cells carrying other CdLS mutations, we utilised two lymphoblastoid cell lines (LCL) previously derived from CdLS patients with heterozygous mutations in NIPBL, Ile1206del^36^ and Arg2298His^37^. These LCLs have significantly more, and larger, 53BP1 foci per cell compared to a WT LCL, in the absence of any exogenous damage (Fig. 6c–e, Supplementary Fig. 8a, b). This suggests that increased DDR signalling and/or impaired DNA repair pathway choice balance may be a common mechanism underlying CdLS caused by BRD4 and NIPBL.

## Discussion

We previously showed that a Y430C-BRD4 mutation, and BRD4 haploinsufficiency, cause a CdLS-like syndrome^8^. The severe developmental phenotypes associated with CdLS are generally thought to be due to aberrant gene regulation. Here, however, we show that BRD4^Y430C^, whilst lowering the affinity of BRD4 to acetylated lysine residues and decreasing its occupancy at enhancers and SEs, causes minor changes in transcription in mESCs, in contrast to the major transcriptional changes caused by the profound loss of BRD4 binding induced by BET inhibitors. Instead, we provide evidence that the BRD4^Y430C^ hypomorph causes increased G2/M checkpoint activation, aberrant DDR signalling and an altered focal accumulation of proteins that promote NHEJ and inhibit HR—53BP1 and the shieldin complex. Conversely there is a depletion of foci containing HR proteins (Rad51), suggesting a defect in HR. Our results suggest a role for BRD4 in the regulation of DNA repair pathway choice and are consistent with recent results from an siRNA screen in human cell lines that suggest that BRD4 deficiency leads to less repair by HR and more by NHEJ^38^. Whether BRD4 mutation affects repair by HR at specific regions in the genome, or globally, remains to be investigated. For example, different levels of histone acetylation in different chromatin environments—e.g. heterochromatin vs euchromatin—upon DNA damage may recruit different amounts of BRD4^39,40^. Alternatively, as well as interacting with NIPBL, Brd4 has been reported to interact with a number of proteins involved in DNA damage sensing and repair, including Rif1 of the Sheildin complex.^8,41^ We cannot exclude that impaired BRD4 function in BRD4^Y430C^ cells affects the formation of DSBs themselves, for example by preventing the accumulation of R-loops^23,38,42^.

We observed a similar focal accumulation of 53BP1 in cells from CdLS patients with mutations in NIPBL. Could aberrant DDR and DNA repair choice, or perhaps elevated DNA damage per se, therefore account for some of the phenotypes associated with CdLS? Congenital mutation in many different genes involved in cell cycle progression and DNA repair, are—like CdLS—generally associated with intrauterine growth retardation and short stature^43^. Similarly, microcephaly also results from mutation in genes associated with S phase progression (ATR, ATRIP, CtIP—Seckel syndrome; DNA ligase IV—lig4 syndrome; XRCC4—microcephalic primordial dwarfism^44–46^). Clinically, there is strongest phenotypic overlap between CdLS and Rubinstein-Taybi syndromes (RTS)—including arched eyebrows and other shared distinctive facial features. RTS is caused by mutations in p300 or CREBBP. These lysine acetyltransferases have recently been shown to be important for acetylating proteins involved in the DDR and DNA repair^47^. NIPBL and cohesin are also both involved in DNA damage signalling and repair^48^ and CdLS patient cells carrying *NIPBL* mutations display an increased DNA damage sensitivity^49^. Even though we cannot discount that *BRD4* mutation in CdLS cases—Y430C, or heterozygous deletions, cause aberrant transcriptional regulation in cell types other than ESCs, our results suggest that dysregulation of DDR and repair may contribute to the aetiology of CdLS.

## Methods

### Cell culture

Y430C-BRD4 mutant and corresponding wild-type mouse embryonic stem cells (mESCs) were generated by CRISPR Cas9 genome editing in 46 C mESCs as described previously^8^. NIPBL I1206del and R2298H lymphoblastoid cell lines (LCLs) were obtained from patients^36,37^. mESCs were cultured in GMEM medium (GIBCO; 11710035) supplemented with 10% Fetal Calf Serum (FCS), 5% penicillin-streptomycin, 1 mM sodium pyruvate (GIBCO; 11360070), 1X non-essential amino acids (GIBCO; 11140050), 50 µM 2-mercaptoethanol (GIBCO; 31350010), 2 mM L-glutamine and 500U/ml Leukaemia Inhibitory Factor (in house). Lymphoblastoid cell lines (LCLs) were grown in RPMI 1640 medium (GIBCO; 11875093) supplemented with 15% FCS and 2 mM L-glutamine. All cells were grown at 37 °C in a 5% CO_2_ humidified atmosphere.

### ChIP-qPCR

Cells were harvested by trypsinising and fixed with 1% formaldehyde (Thermo Fisher; 28906) in media (25 °C, 10 min). This reaction was quenched with 0.125 M glycine for 5 min. ChIP-qPCR was performed as described previously^8^ (see Table 1 for antibodies). DNA was purified using the QIAquick PCR Purification kit (Qiagen, 28104). Input samples were diluted to 1%, and all samples diluted a further 10-fold, in ddH_2_0. SYBR-green based qPCR reactions were performed in a final volume of 20 µl containing diluted ChIP DNA, SYBR select master mix (ThermoFisher Scientific; 4472908) and 0.25 µM/L of each primer (see Table 1). Concentration of IPs are relative to 1% input.

**Table 1:** Key resources.

Antibodies	Source	Identifier
BRD4	Bethyl	Cat# A301-985A-M
53BP1	Novus	Cat# NB100-304
Normal Rabbit IgG	Santa Cruz	Cat# sc-2025
CHK1	Abcam	Cat# ab47574
CHK1-p	Cell signalling technologies	Cat# 2348
Lamin B	Santa Cruz	Cat# sc-374015
MAD2L2	Abcam	Cat# ab180579
RIF1	A kind gift from Sara Buonomo	Rabbit anti–mouse Rif1 serum 1240^52^
RAD51 for IF	Calbiochem	Cat# PC130
RAD51 for WB	Santacruz	Sc-8349
γH2AX	Merck-Millipore	Cat# 05-636
γTubulin	Abcam	Ab11316
Goat anti-Rabbit IgG, secondary, Alexa Fluor 488	Invitrogen	Cat# A11034
Donkey anti-Rabbit IgG, secondary, Alexa Fluor 586	Invitrogen	Cat# A10042
**Primers**	**Forward**	**Reverse**
ChIP-qPCR		
Sox2 SE	TAGAGGAAGGAGCTGGAGGA	AAGGAAAGAAGGAGGGACGG
Klf4 SE	CACAATGCCAGCTATGCGAT	TCCTGCCCAAATGTGAGGAT
Nanog SE	GTGAAGGTAGTTTGCTGGGC	GGTCCTTTCCCACCCTCTAC
Oct4 SE	CCTTCGTTCAGAGCATGGTG	GAGCCTACCCTGAACTTCCC
Expression		
Klf4	GTGCAGCTTGCAGCAGTAAC	AGCGAGTTGGAAAGGATAAAGTC
Myc	CCCTAGTGCTGCATGAGGA	CGTAGTTGTGCTGGTGAGTG
Oct4	CGAGAACAATGAGAACCTTC	CCTTCTCTAGCCCAAGCTGAT
Nanog	TGGTCCCCACAGTTTGCCTAGTTC	CAGGTCTTCAGAGGAAGGGCGA
Software and algorithms		
Bowtie2	Langmead and Salzberg, 2012	http://bowtie-bio.sourceforge.net/bowtie2/index.shtml
MACS2		https://github.com/taoliu/MACS
FACSDiva software	BD Bisoscience	
TopHat	Trapnell et al., 2012	https://ccb.jhu.edu/software/tophat/index.shtml
Cufflinks	Trapnell et al., 2012	http://cole-trapnell-lab.github.io/cufflinks/
Deeptools2	Ramirez et al., 2016	http://deeptools.readthedocs.io/en/latest/index.html
SAMtools	Li et al., 2009	http://samtools.sourceforge.net/

Deposited Data		
BRD4-WT ChIP-seq	This paper	GSE130659
BRD4-Y430C ChIP-seq	This paper	GSE130659
WT 4sU-seq	This paper	GSE130659
Y430C 4sU-seq	This paper	GSE130659
WT Spike-in RNAseq	This paper	GSE130659
Y430C Spike-in RNAseq	This paper	GSE130659
	This paper	GSE130659

### ChIP-seq

ChIP was carried out as above. After purification, DNA was eluted in 20 μl and libraries were prepared for ChIP and input samples as previously described^50^. Samples were sequenced at BGI (Hong Kong; 50-bp single-end reads) using the HiSeq 4000 system (Illumina). Fastq files were quality controlled using FastQC and mapped to the mm9 genome using Bowtie2 (parameters: default). Sam files were converted to bam files and sorted using SamTools. Homer was used to make tagdirectories (makeTagDirectory, parameters: –unique, fragLength 150) and bedgraphs (makeUCSCfile, parameters: default). For visualisation of BRD4 data, bedgraphs were uploaded to the genome browser UCSC. Peak calling was carried out using MACS2; duplicates were filtered (filterdup, parameters:—keep-dup=1), peaks called (callpeaks, parameters: -B—nomodel -p 1e-5) and differential peaks were found (bdgdiff, parameters: -g 60 -l 250).

deepTools2 was used to make heatmaps; score files were made across specific genomic regions (computeMatrix, parameters: scale-regions scale regions –b 500 –a 500 –bs 50 –bl mm9 blacklist) and these were used to plot heatmaps (plotHeatmap, parameters:—colormap RdBluYl reverse).

### JQ1 treatment

1 mM BRD4 inhibitor JQ1 + , or its inactive form JQ1- (Merck; 500586) (diluted in DMSO), were added to mESC media at a final concentration of 300 nM. JQ1 + /-. WT and Y430C mESCs were incubated at 37 °C with JQ1 + /- supplemented media for 48 h. Total RNA was extracted from cells using the RNeasy Plus Mini Kit (Qiagen; 74134) and 1 µg RNA was used for cDNA synthesis with SuperScript II Reverse Transcriptase (ThermoFisher Scientific; 18064-014) as per manufacturer’s instructions. cDNA was diluted 1:500 for qPCR analysis. qPCR reactions were performed as above (see Table 1 for primers). Concentration of JQ1 + cDNA was calculated relative to JQ1- (arbitrarily set to 1).

### RT-PCR

RNA was extracted from cells using the RNeasy Mini Kit (Qiagen; 74104) using spin technology, with an additional on-column DNA digestion using the RNase-Free DNase Set (Qiagen; 79254). cDNA was synthesised from 1 μg RNA using SuperScript II Reverse Transcriptase (ThermoFisher Scientific; 18064-014) as per manufacturer’s instructions. cDNA was diluted 1 in 25 for qPCR analysis. SYBR-green based qPCR reactions were performed in a final volume of 20 μl containing diluted cDNA, SYBR select master mix (ThermoFisher Scientific; 4472908) and 0.5 μM/l of region specific intron-spanning primer pairs.

### 4sU-seq

4sU RNA was generated and isolated as described previously^51^, with the following changes: cells were incubated at 37 °C with 4sU-supplemented medium for 20 min. The reaction was incubated with Biotin-HPDP with rotation for 1.5 h at RT. For recovery of biotinylated 4sU-RNA, 1 µl of streptavidin beads was added per μg of RNA. Columns were washed using 900 µl washing buffer and RNA was eluted by 2 sequential additions of 100 µl Elution Buffer (100 mM DTT) to the column and eluates combined. RNA was further purified using the RNAeasy MinElute Clean-up kit (Qiagen; 74204) according to the manufacturer’s guidelines, eluting in 20 µl water. 1 μl of 4sU-labelled RNA was quality-checked by running on a 2100 Bioanalyzer Instrument (Agilent).

To make 4sU sequencing libraries, 4sU labelled RNA was first depleted of rRNA using the Low Input Ribominus Eukaryotic System V2 (ThermoFisher Scientific; A15027) as per the manufacturer’s instructions. 600 ng of 4sU labelled RNA was used as input, and eluted in 5 µl RNase free water. All of the resulting rRNA free RNA was used to prepare 4sU sequencing libraries, using NEBnext Ultra Directional RNA library prep kit of Illumina (NEB; E7420). RNA fragmentation was carried out at 94 °C for 15 min, as suggested for intact RNA. Libraries were indexed with Multiplex Oligos for Illumina^®^ (Index Primers Set 1) (NEBnext; E7335) and amplified by PCR for 13 cycles. Library concentration and correct size distribution was confirmed on the Agilent 2100 Bioanalyser with the DNA HS Kit. Libraries were sequenced at BGI (Hong Kong; 100-base paired-end reads) using the HiSeq 4000 system (Illumina).

Fastq files were quality controlled using FastQC and mapped to the mm9 genome using tophat (parameters:—library-type fr-firststrand -r 200). Homer was used to make tagdirectories (makeTagDirectory, parameters: -unique -sspe -flip -fragLength 150), and to make bedgraphs for visualisation on UCSC (makeUCSCfile, parameters: -strand separate -style rnaseq). Cufflinks was used for peak calling; transcripts were assembled for individual experiments (cufflinks, parameters: –m 200 –library-type fr) and both replicates of WT and Y430C were combined to form one assembly (cuffmerge, parameters: default). Differentially expressed peaks were determined from this assembly using cuffdiff (Cuffdiff. Parameters: default).

Heatmaps were generated as above.

### Spike-in RNA-seq

S2 cells were cultured in Schneider’s Drosophila Medium (Invitrogen; 11720-034), supplemented with 10% heat-inactivated FCS and 5% penicillin-streptomycin. Cells were passaged once they reached a density of ~2 × 10^7^ cells/ml and seeded at a density of ~4 × 10^6^. Cells were grown at 28 °C in a 5% CO_2_ humidified atmosphere. Cells were frozen at a density of ~1 × 10^7^ cells/ml in 45% conditioned Schneider’s Drosophila Medium media (containing 10% FCS), 45% fresh Schneider’s Drosophila Medium supplemented with 10% FCS, and 10% DMSO, and stored in liquid nitrogen.

mESCs and S2 cells were harvested and counted. 0.2 million S2 cells were mixed with 10 million mESCs, and RNA was extracted using the RNeasy Mini Kit (Qiagen; 74104) using spin technology, with an additional on-column DNA digestion using the RNase-Free DNase Set (Qiagen; 79254). RNA was depleted of rRNA and RNA-seq libraries prepared as for the 4sU-seq.

### Growth assay

WT and Y430C mESCs were each seeded in 4 wells of a 6 well plate (1 × 10^4^ cells/well). WT and Y430C cells from 1 well were trypsinised and counted at 24, 48, 72 and 96 h post seeding. Counting was carried out manually using a haemocytometer. The addition of trypan blue dye allowed for the exclusion of dead cells.

### Flow cytometry

2 million mESCs were fixed in 70% ethanol (in PBS) at 4°C for 1 h. Fixed cells were centrifuged at 300 *g* at 4°C for 5 min, washed twice with PBS and resuspended in 500 µl PBS. 20 µg RNase A was added and cells were incubated at 37°C for 10 min. Cells were stained with propium iodide at a final concentration of 50 µg/ml. Acquisition was carried out on a BD LSRFortessa cell analyser, collecting 25,000 events per sample. Results were analysed using BD FACSDiva 8.0.1 and gated cells were manually categorised into cell cycle stages G0/G1, S and G2/M.

### NCS treatment and CHK-1 protein Western blots

Cells were incubated with mESC media supplemented with neocarzinostatin (Sigma; N9162) (NCS), to a final concentration of 25 ng/ml, for 15 min at 37 °C. Cells were then washed with PBS and fresh, non-supplemented media was added. Protein was either extracted straight away, or after incubation at 37 °C for varying lengths of time. Ice-cold RIPA buffer (150 mM sodium chloride; 1.0% NP-40; 0.5% sodium deoxycholate; 0.1% SDS; 50 mM Tris, pH 8.0) was added to plates (1 ml per 10^7^ cells) and cells were scraped and transferred into pre-chilled microcentrifuge tubes. Tubes were shaken at 4 °C for 30 min before centrifugation at 20,000 x *g* for 15 min. Supernatant was retained and quantified. For Western blot analysis, equal amounts of protein were boiled in 1X NuPage LDS buffer (ThermoFisher Scientific, NP0008) with 1X NuPage reducing agent (ThermoFisher Scientific; NP0004) for 5 min and separated on a 3–8% tris-acetate gel (ThermoFisher Scientific; EA0375BOX). Following electrophoresis, proteins were transferred to nitrocellulose membranes (ThermoFisher Scientific) and immunoblotted with primary antibodies overnight at 4 °C. Membranes were washed 3 X TBST and probed with HRP-conjugated secondary antibody for 1 h at RT. After 3 more washes in TBST, membranes were incubated with SuperSignal™ West Femto Maximum Sensitivity Substrate (ThermoFisher Scientific; 34095) for 5 min and imaged using ImageQuant™ LAS 4000 (GE Healthcare).

### Immunofluorescence

mESCs for immunofluorescence experiments were cultured on gelatinised coverslips and LCLs were grown in suspension. LCLs were harvested and resuspended in PBS to 1.8 × 10^5^ cells/ml. 500 µl of cell suspension was added to a Shandon™ Single Cytofunnel™ (ThermoFisher Scientific; 5991040), with a microscope slide attached. Slides were centrifuged at 800 rpm for 5 min, after which the LCLs had attached to the slide. All cells were fixed in 4% paraformaldehyde for 10 min and washed 3 × 3 min in PBS. Cells were then permeabilised in 0.5% Triton in PBS for 10 min and washed 3 × 3 min in PBS. Cells were blocked in 1% BSA in PBS for 30 min at RT, incubated with primary antibody diluted in 1% BSA for 1 h at RT and washed 3 × 3 min in PBS. Cells were next incubated with secondary antibody (see Table 1) diluted in 1% BSA for 45 min at RT, washed 3 × 3 min in PBS, incubated with DAPI in PBS (250 ng/ml) for 2 min, and washed 3 × 3 min in PBS. Coverslips were mounted on slides in Vectashield (Vector; H1000) mounting medium for fluorescence.

All slides were viewed, and foci counted, using epifluorescence microscopes. Images were taken using confocal microscopy.

### Laser microirradiation of U2OS cells

U2OS cells (ATCC^®^ HTB-96™) were grown on glass coverslips and incubated with 10 μM BrdU (Sigma Aldrich, #B9285) for 24 h prior to microirradiation. Microirradiation was induced with a 405 nm laser diode (3 mW) focused through a 63x/1.4 oil objective on a Zeiss LSM710 confocal microscope using the following laser settings: 40% power, 50 iterations, scan speed 12.6 μsec/pixel. Cells were fixed either 10 min or 2 h after laser irradiation using 2% PFA and immunofluorescence was performed as described in the immunofluorescence section using γH2AX antibody (Merck-Millipore, #05-636) as positive control of DNA damage induction. Image acquisition was performed on a Leica DMI6000 epifluorescence microscope using a Plan-Apochromat 40x/1.3 oil objective.

### Reporting summary

Further information on research design is available in the Nature Research Reporting Summary linked to this article.

## Supplementary information

Supplementary Information

Reporting Summary

## Data Availability

Data supporting the findings of this study have been deposited in GEO with accession number GSE130659. All data is available from the authors upon reasonable request. Source data are provided with this paper.

## Source data

Source Data

## Code availability statement

Custom script for analysis of foci area is deposited here: https://github.com/IGMM-ImagingFacility/Boumendil2020_BRD4paper; 10.5281/zenodo.4596974.
