# Supplementary material for: Cornelia de Lange syndrome-associated mutations cause a DNA damage signalling and repair defect
Source: Nat Commun. 2021 May 25;12:3127. doi: 10.1038/s41467-021-23500-6 (PMC8149872; doi:10.1038/s41467-021-23500-6)
Supplement: Supplementary file 1 — Supplementary Information [file 41467_2021_23500_MOESM1_ESM.pdf]

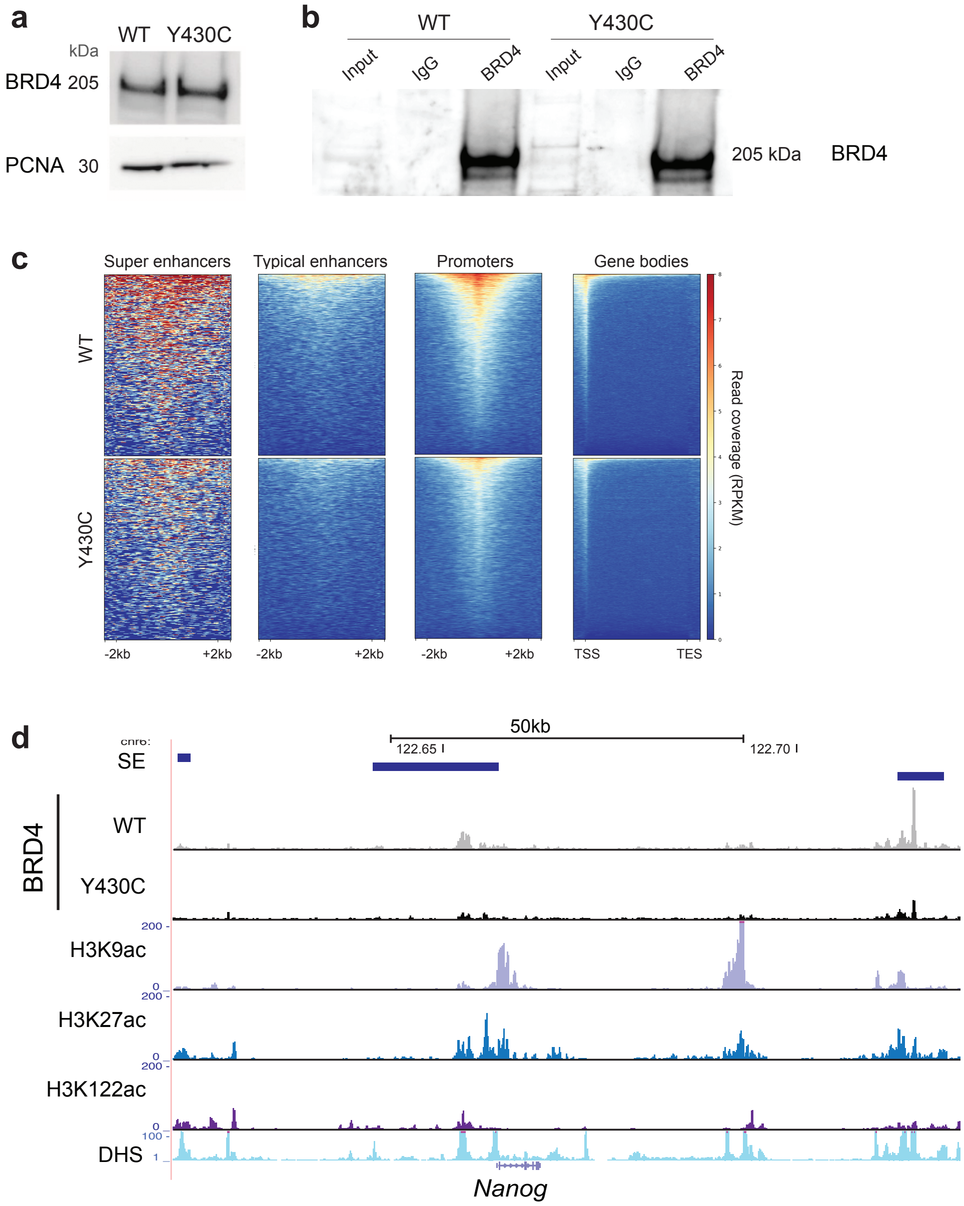

Figure S1. Replicate BRD4 ChIP-seq data for an independent BRD4Y430C clone. a) Immunoblot for BRD4 and PCNA from lysates of wild-type (WT) and BRD4^{Y430C} mESCs. b) Immunoprecipitates for BRD4 and IgG from WT and BRD4^{Y430C} mESCs immunoblotted for BRD4. 1% of input is also shown. c)Heatmaps show enrichment of WT and BRD4Y430C over super enhancers, typical enhancers, promoters and gene bodies. d) UCSC browser screenshot showing reads per 10 million (RP10M) over regions of the *Nanog* locus (SE = super-enhancer, TE = typical enhancer, Gene = transcribed region) for BRD4 ChIP-seq in WT and BRD4^{Y430C} mESCs that are an independent clone from that used in Figure 1. Below are shown previously published ChIP-seq data for H3K27ac (ENCSR000CDE), H3K9ac (ENCSR000CGS), H3K122ac (GSE66023) and DNase I hypersensitivity.

Supplementary Figure 1

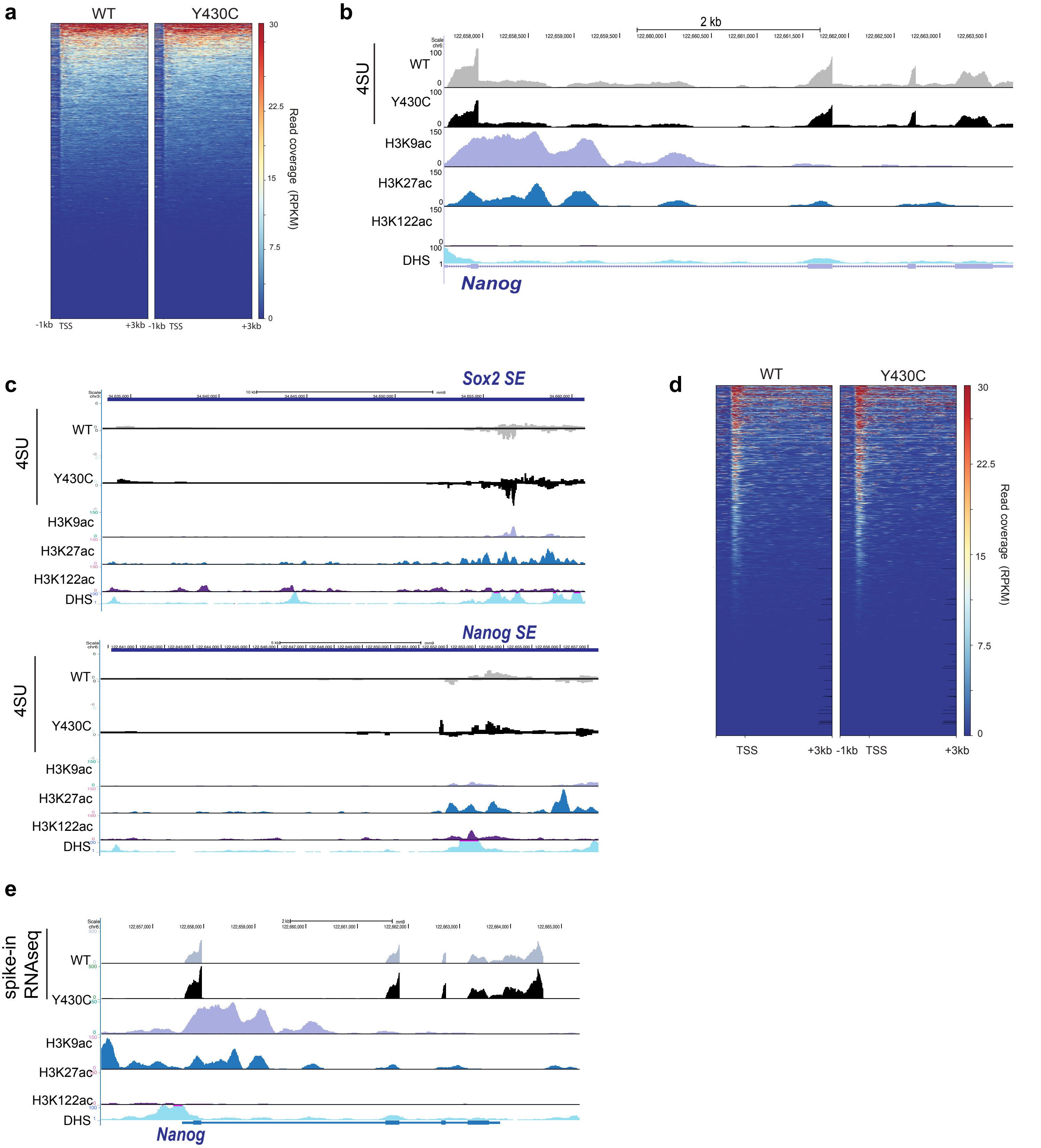

Figure S2. Replicate 4sU-seq data. Data from a Brd4Y430C clone that is independent of that shown in main Figure 2. a) Heatmaps show enrichment of WT and Y430C 4sU-seq over transcribed regions (mm9_refseq). b) UCSC browser screenshot showing reads per 10 million (RP10M) over the Nanog transcribed region for 4sU-seq in wild-type (WT) and *BRD4*^{Y430C} mESCs. Below are shown published ChIP-seq data for H3K27ac (ENCSR000CDE), H3K9ac (ENCSR000CGS), H3K122ac (GSE66023) and DNase I hypersensitivity. c) As in (b) but for the *Sox2*, *Klf4* and *Nanog* SEs d) Heatmaps show enrichment of WT and *BRD4*^{Y430C} 4sU-seq normalised to spike-in Drosophila RNA over transcribed regions (mm9_refseq). e) UCSC browser screenshot showing RP10M normalised to spike-in Drosophila RNA over *Nanog* in WT and Y430C mESCs.

a

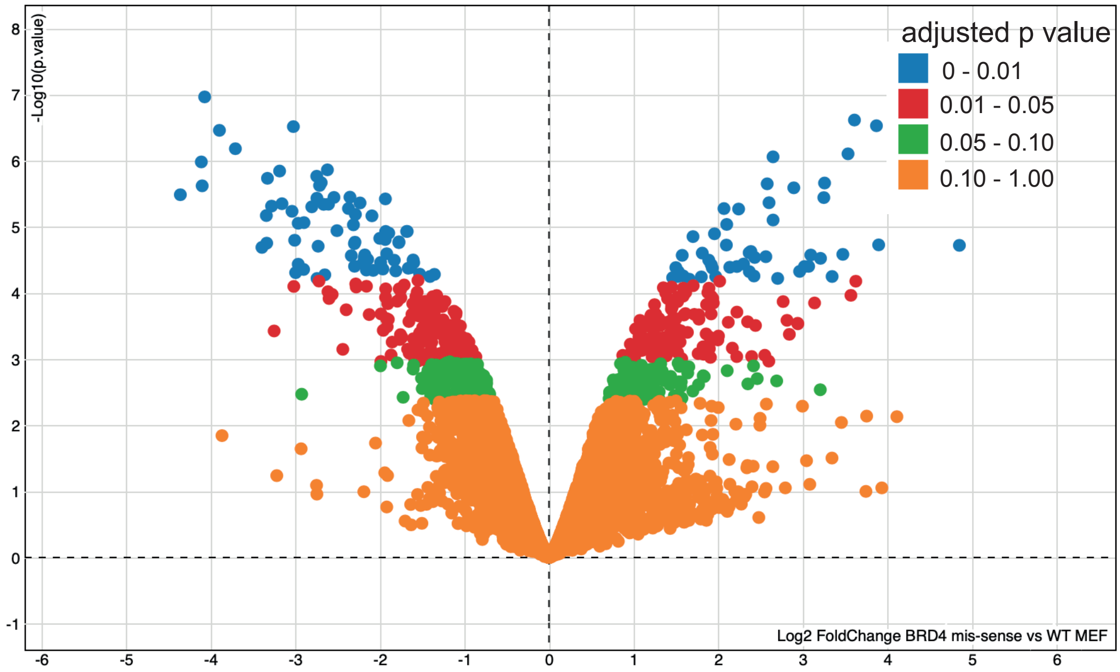

b

	SYMBOL	logFC	AveExpr	t	P.Value	adj.P.Val	B	log10p	adjp_group
1	Crabp1	-4.070400001	11.69007546	-19.30791352	1.08e-7	0.001323302	7.748659342	6.96657624451305	[0.00,0.01)
2	Lyz2	3.610750189	10.09867377	17.31899712	2.42e-7	0.001323302	7.199213406	6.61618463401957	[0.00,0.01)
3	Lyz1	3.870895845	10.81727679	16.85760963	2.95e-7	0.001323302	7.056140569	6.53017798402184	[0.00,0.01)
4	Sparcl1	-3.021436733	8.947440919	-16.78636283	3.04e-7	0.001323302	7.033469599	6.51712641639125	[0.00,0.01)
5	Sned1	-3.895825503	11.40415054	-16.49468607	3.46e-7	0.001323302	6.938990994	6.46092390120722	[0.00,0.01)
6	Ramp2	-3.707171012	10.17793815	-15.11831788	6.56e-7	0.002081125	6.454338707	6.18309616062434	[0.00,0.01)
7	C1qc	3.534760927	7.912876288	14.75513218	7.84e-7	0.002081125	6.314831809	6.10568393731556	[0.00,0.01)
8	C1qa	2.648440926	8.891789689	14.54385529	8.71e-7	0.002081125	6.231255577	6.05998184499234	[0.00,0.01)
9	Sfrp2	-4.109752687	13.43644325	-14.19918468	0.00000104	0.002203837	6.090937462	5.98296666070122	[0.00,0.01)
10	Thbs4	-2.619336107	10.90726146	-13.6666256	0.00000137	0.0024878	5.863970784	5.86327943284359	[0.00,0.01)
11	Dio3	-3.184472358	13.03731257	-13.58535669	0.00000143	0.0024878	5.828204752	5.84466396253494	[0.00,0.01)
12	Ndufa4l2	-2.745242387	10.75142414	-13.26124067	0.00000171	0.002541299	5.682459441	5.76700388960785	[0.00,0.01)
13	Clec11a	-3.327893586	9.781155687	-13.12668194	0.00000184	0.002541299	5.620459192	5.73518217699046	[0.00,0.01)
14	H19	-2.692950095	12.69168488	-12.85594024	0.00000214	0.002541299	5.492967541	5.66958622665081	[0.00,0.01)
15	Perp	3.257383823	10.28621057	12.8284076	0.00000217	0.002541299	5.479793184	5.66354026615147	[0.00,0.01)
16	Gjb2	2.579125083	9.889950439	12.783662	0.00000223	0.002541299	5.458298729	5.65169513695184	[0.00,0.01)
17	Atp1b1	-2.711861057	11.03209406	-12.68324145	0.00000236	0.002541299	5.409679395	5.62708799702989	[0.00,0.01)
18	Egfl6	-4.099634787	8.438297101	-12.65642875	0.00000239	0.002541299	5.396608142	5.62160209905186	[0.00,0.01)
19	Tnfrsf11b	2.893672051	10.83374641	12.53979259	0.00000256	0.002574246	5.339302948	5.59176003468815	[0.00,0.01)
20	Clu	-4.357733587	12.05338961	-12.12447571	0.00000326	0.002879049	5.129224896	5.48678239993206	[0.00,0.01)
21	Arhgap20	-2.352728553	9.451714777	-11.98228268	0.00000355	0.002879049	5.055069354	5.44977164694491	[0.00,0.01)
22	Ebf3	-2.541775513	10.30087847	-11.96948272	0.00000358	0.002879049	5.048336697	5.44611697335613	[0.00,0.01)
23	Angptl7	3.248775933	9.692006158	11.96126246	0.0000036	0.002879049	5.044007886	5.44369749923271	[0.00,0.01)
24	Agtr1a	-2.743397978	10.55088353	-11.92510172	0.00000368	0.002879049	5.024918707	5.43415218132648	[0.00,0.01)
25	Irak3	-1.933915973	7.817320988	-11.88573872	0.00000377	0.002879049	5.004051955	5.42365864979421	[0.00,0.01)

Figure S3. Analysis of gene expression between WT and Brd4^{Y430C} mutant MEFs. Analaysis of gene expression data from MEFs derived from WT and *Brd4^{Y430C}* mouse embryos and analysed by microarray. Data are from ref 8. a) Scatter plot showing log2 fold expression change between *Brd4^{Y430C}*mutant and wild-type MEFs against adjusted p value (log10). Colour coding indicates adjusted p value group. b) Top 25 mis-regulated genes identified in (a).

DE expression analysis was carried out using the limma bioconductor package in R to find genes with an absolute LogFC > 0. A linear model was fitted to each probe. Then empirical Bayes moderation was applied to the linear model fit to compute moderated t-statistics, moderated F-statistic, and log-odds of differential expression .The Benjamini & Hochberg (alias FDR) method was used to correct the p-values for multiple testing.

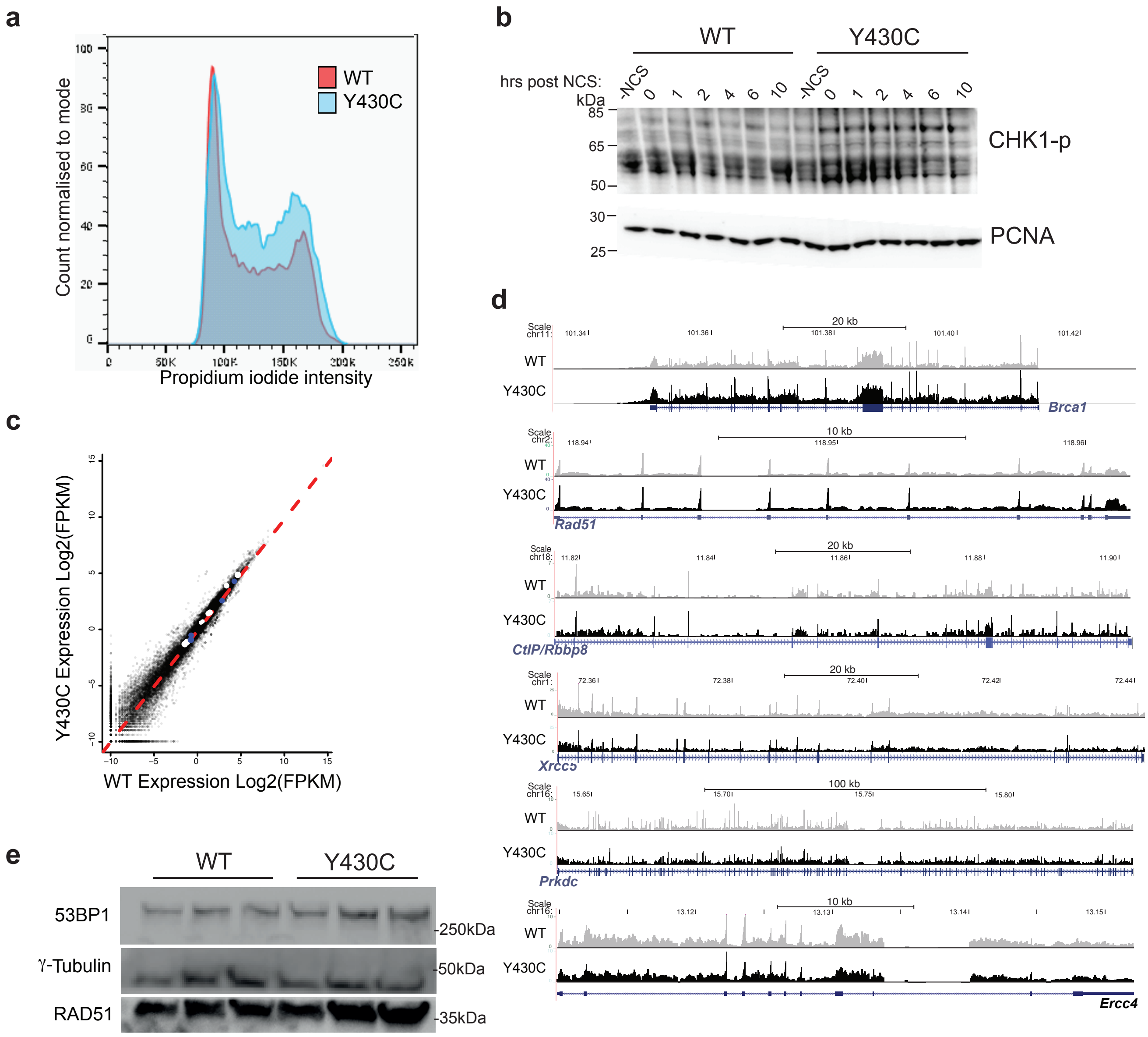

Figure S4. Replicate of G2/M checkpoint activation and analysis of transcription of DNA damage related genes in Y430C mESCs. a) Overlaid cycle profiles for WT and BRD4^{Y430C} cell, as determined by flow cytometry after propidium iodide staining. b) Immunoblot using antibodies against CHK1-p and PCNA after treatment of WT and Brd4Y^{430C} mESCs with NCS at various times (hrs) after recovery. Similar result was obtained in an independent experiment and is shown in figure 3. c) Scatter plot of the 4SU-seq data from WT and BRD4^{Y430C} cells, highlighting (white) genes involved in HR (CtIP, BRCA1, BRCA2, Rad51, Rad54) and (blue) NHEJ (Ku70, Ku80, XRCC4, Artemis, Lig4). Red dashed line shows best fitted line. d) UCSC browser screenshot showing reads per 10 million (RP10M) for 4sU-seq from WT and Y430C cells over the transcribed regions of DNA damage response genes Brca1, Rad51, CtIP, Xrcc5 and Ercc4 Genome co-ordinates (Mb) are from the mm9 assembly of the mouse genome. e) Immunoblot of protein lysates from WT and Brd4Y^{430C} mESCs stained with antibodies detecting 53BP1, RAD51 and γ-tubulin.The three different columns of WT or Y430C are cell extracts from three independent experiments.

Supplementary figure 4

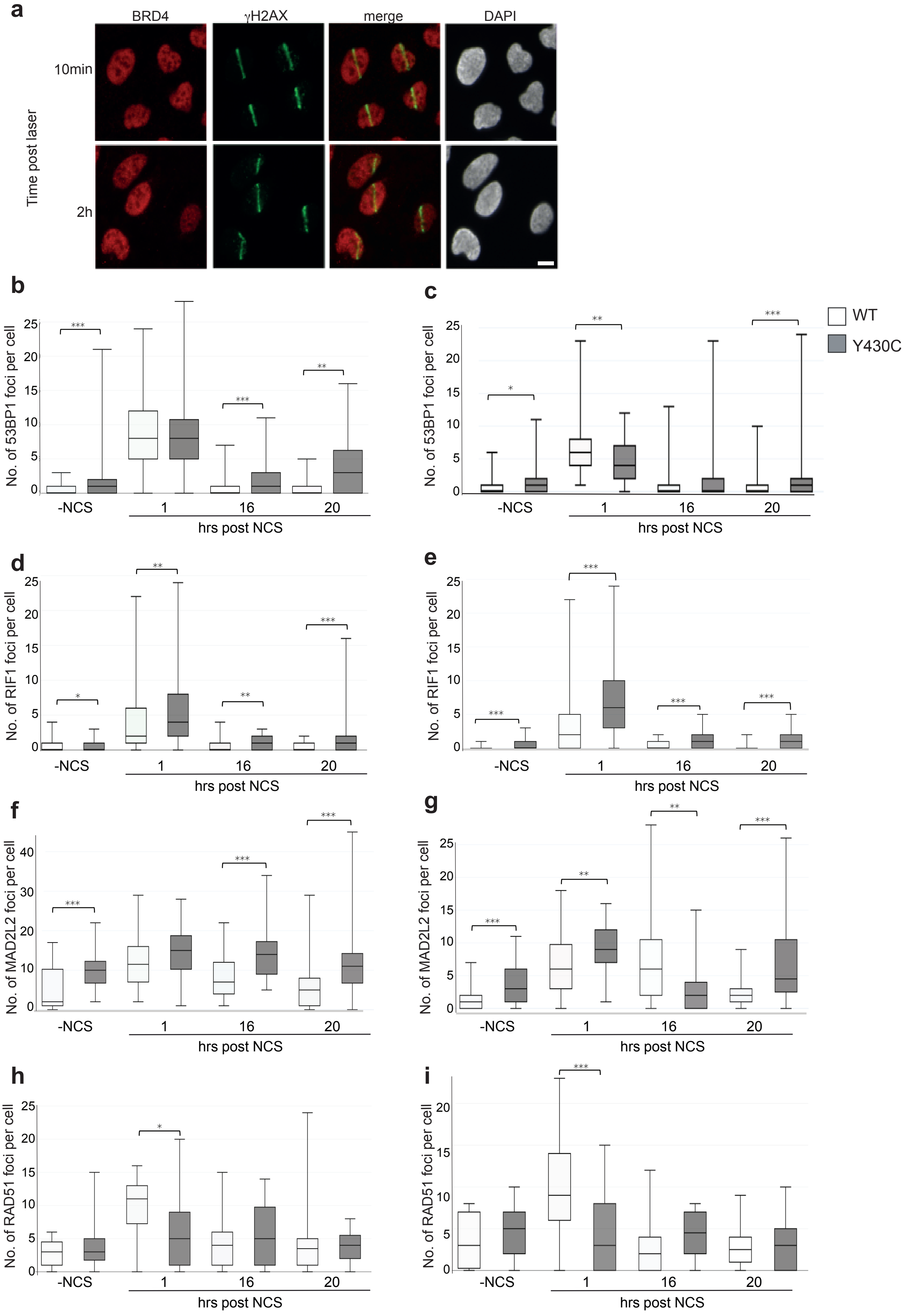

Figure S5. No accumulation of BRD4 at damage sites and replicates of 53BP1, RIF1, MAD2L2 and RAD51 foci after DSB induction in WT and Y430C mESCs. a) Immunofluorescence for BRD4 and γH2AX in the DAPI-stained nuclei of U2OS cells presensitized with 10μM BrdU for 24h and irradiated with 405nm laser shows no BRD4 accumulation at damage sites 10min (upper line) or 2h (lower line) after irradiation. Scale bar: 10μM. Similar results were obtained in three independent experiments. Box-plots show number of b&c) 53BP1, d&e) RIF1, f&g) MAD2L2, h&i) RAD51 foci per cell in WT and Y430C cells after treatment with NCS. Horizontal lines within boxes show medians, boxes are inter-quartile ranges and whiskers are range. P-values were calculated with Mann-Whitney one-sided U test. * < 0.05, ** < 0.01, *** < 0.001. 50 cells have been examined per condition. Data are additional replicates of the data in Figures 4 and 5.

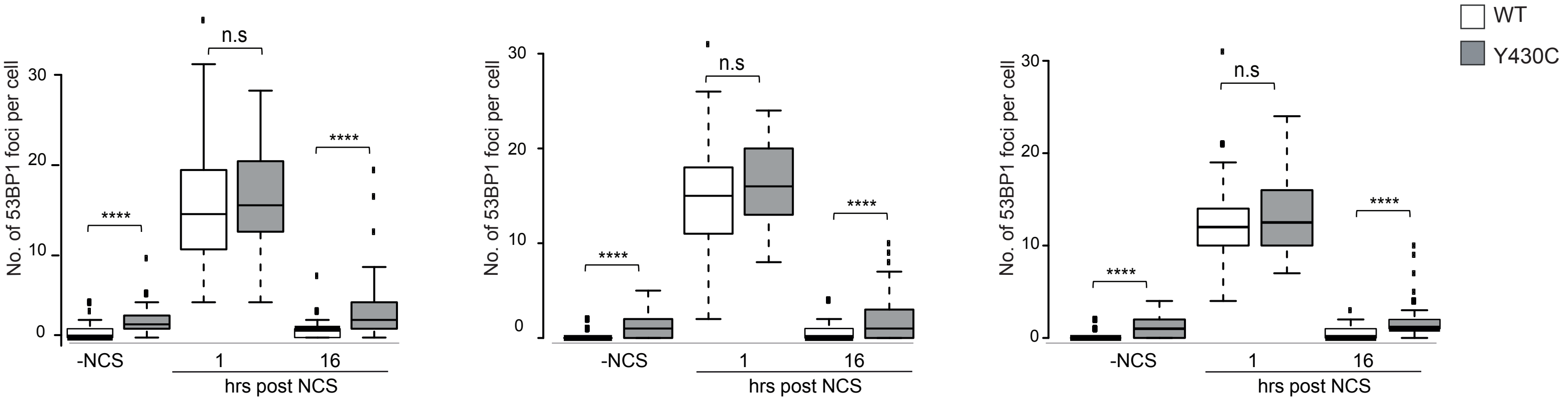

Figure S6. Increased number of 53BP1 foci after DSB induction in diploid Y430C mESCs.
Box-plots show number of 53BP1 foci per nucleus in WT and Y430C mESCs upon treatment with NCS and after recovery periods up to 16hrs in one experiment each. Horizontal lines within boxes show medians, boxes are inter-quartile ranges and whiskers are range. P-values were calculated with Mann-Whitney one-sided U test. N.s: non significant, p-value>0.01 ****: p-value<0.00001. 50 cells have been examined per condition.

Supplementary figure 6

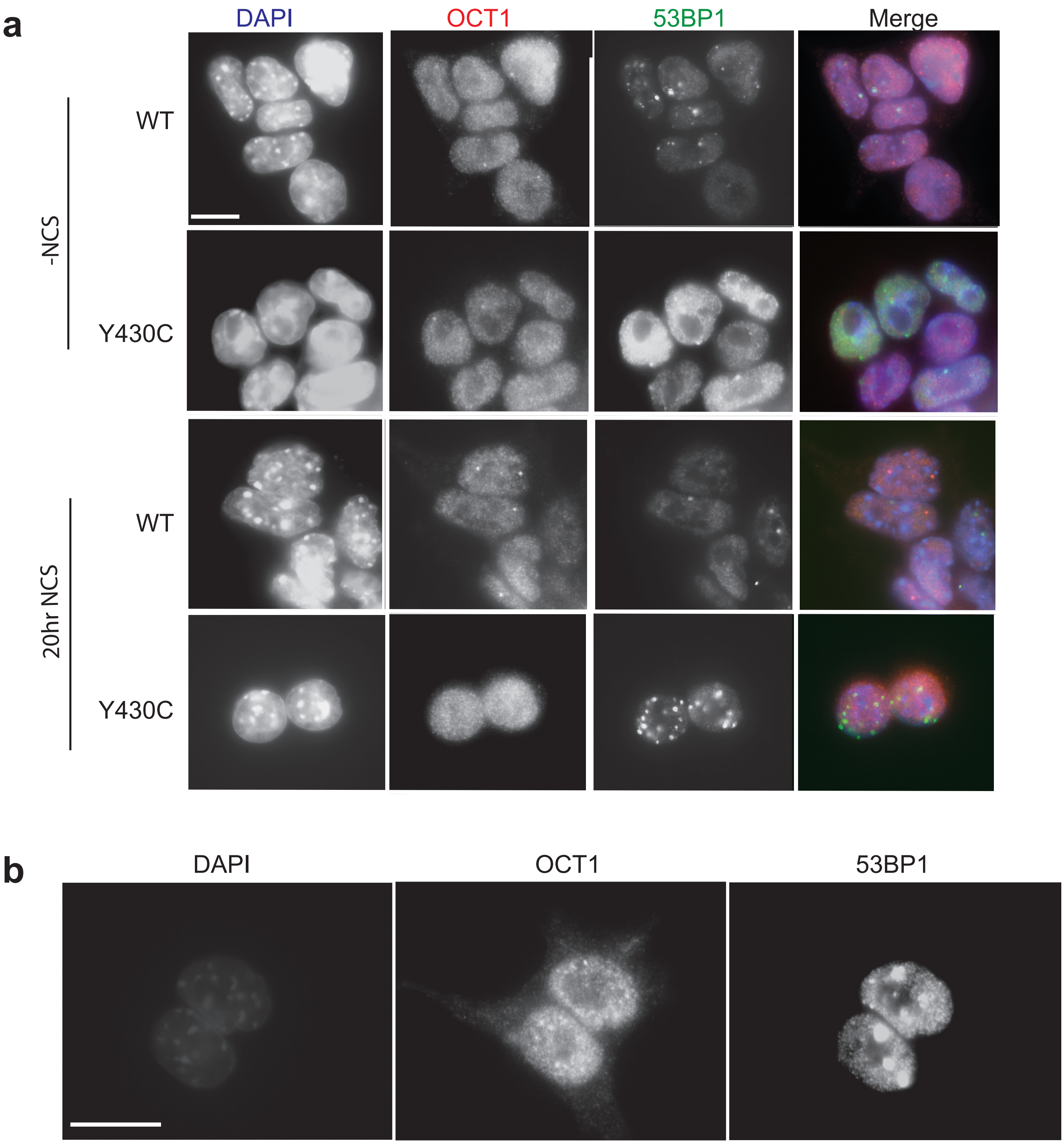

Figure S7. 53BP1 and Oct1 (OPT) foci do not co-localise. a) Immunofluorescence for 53BP1 (green in merge) and Oct1 (red in merge) in the DAPI-stained nuclei of wild-type and BRD4^{Y430C} mESCs before (-NCS) and 20 hours after treatment with NCS. Scale bar: 10 μm b) Immunofluorescence for 53BP1 and Oct1 in the DAPI-stained nuclei of daughter G1 BRD4^{Y430C} cells. Scale bar: 10μm
Similar results were obtained in an independent experiment.

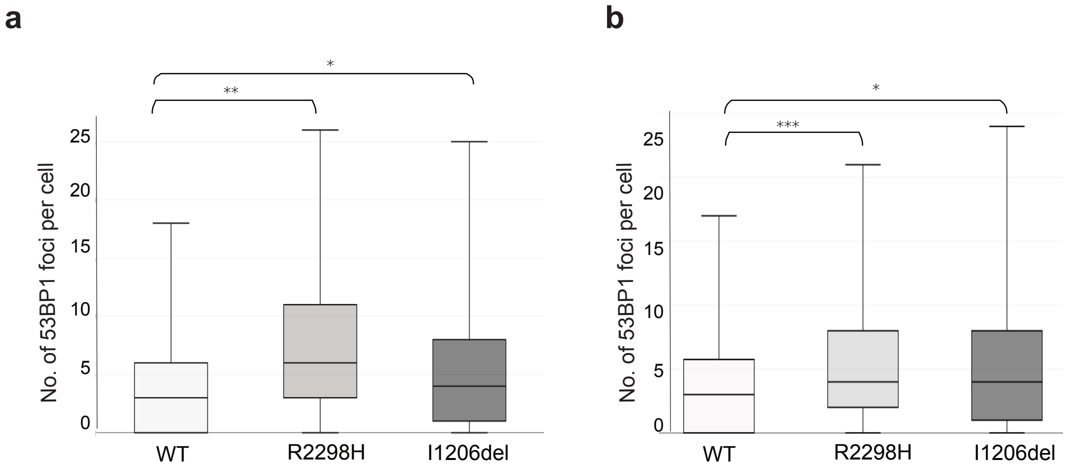

Supplementary Figure 8. Replicates of number of 53BP1 foci in WT and NIPBL mutant CdLS lymphoblastoid cell lines. a&b) Box-plots show number of 53BP1 foci per cell in WT, NIPBL+/R2298H and NIPBL+/I1206del LCLs. Horizontal lines within boxes show medians, boxes are inter-quartile ranges and whiskers are range. P-values were calculated with Mann-Whitney one-sided U test. * < 0.05, ** < 0.01, *** < 0.001. 50 cells have been examined per condition.

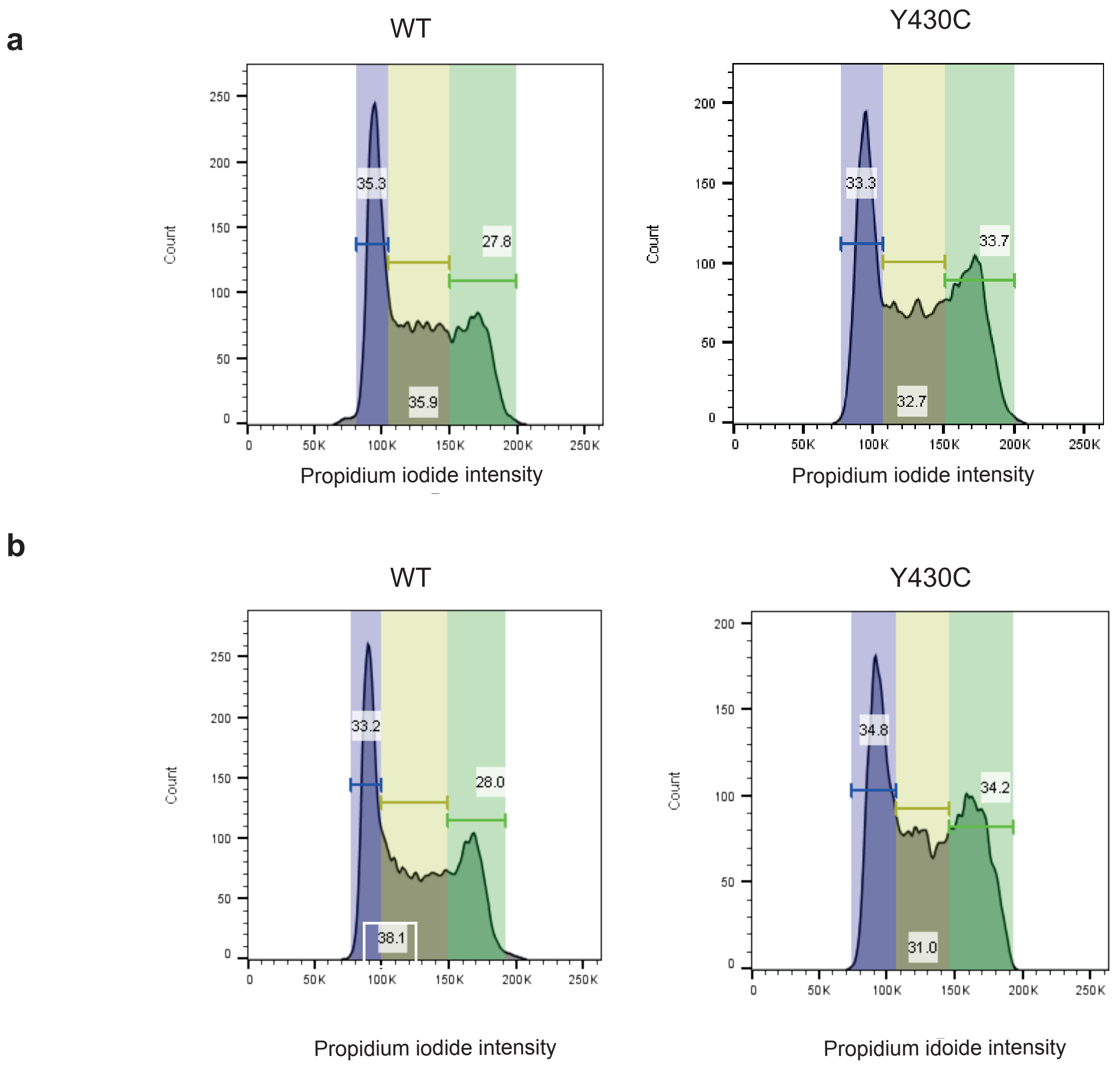

Figure S9. Gating strategy for cell cycle analysis Gating strategy for flow cytometry analysis of cell cycle in WT and Y430C mESCs of profiles shown in Figure 3b (a) and Supplementary Figure 4a (b)
