# Supplementary material for: Cornelia de Lange syndrome-associated mutations cause a DNA damage signalling and repair defect
Source: Nat Commun. 2021 May 25;12:3127. doi: 10.1038/s41467-021-23500-6 (PMC8149872; doi:10.1038/s41467-021-23500-6)
Supplement: Supplementary file 2 — Reporting Summary [file 41467_2021_23500_MOESM2_ESM.pdf]

nature research

Corresponding author(s): W.A Bickmore C. Boumendil

Last updated by author(s): Apr 9, 2021

# Reporting Summary

Nature Research wishes to improve the reproducibility of the work that we publish. This form provides structure for consistency and transparency in reporting. For further information on Nature Research policies, see Authors & Referees and the Editorial Policy Checklist .

## Statistics

For all statistical analyses, confirm that the following items are present in the figure legend, table legend, main text, or Methods section.

| n/a | Confirmed | |
|---|---|---|
| ☐ | ☒ | The exact sample size (*n*) for each experimental group/condition, given as a discrete number and unit of measurement |
| ☐ | ☒ | A statement on whether measurements were taken from distinct samples or whether the same sample was measured repeatedly |
| ☐ | ☒ | The statistical test(s) used AND whether they are one- or two-sided<br>*Only common tests should be described solely by name; describe more complex techniques in the Methods section.* |
| ☒ | ☐ | A description of all covariates tested |
| ☒ | ☐ | A description of any assumptions or corrections, such as tests of normality and adjustment for multiple comparisons |
| ☐ | ☒ | A full description of the statistical parameters including central tendency (e.g. means) or other basic estimates (e.g. regression coefficient) AND variation (e.g. standard deviation) or associated estimates of uncertainty (e.g. confidence intervals) |
| ☐ | ☒ | For null hypothesis testing, the test statistic (e.g. *F*, *t*, *r*) with confidence intervals, effect sizes, degrees of freedom and *P* value noted<br>*Give P values as exact values whenever suitable.* |
| ☒ | ☐ | For Bayesian analysis, information on the choice of priors and Markov chain Monte Carlo settings |
| ☒ | ☐ | For hierarchical and complex designs, identification of the appropriate level for tests and full reporting of outcomes |
| ☒ | ☐ | Estimates of effect sizes (e.g. Cohen's *d*, Pearson's *r*), indicating how they were calculated |

*Our web collection on statistics for biologists contains articles on many of the points above.*

## Software and code

Policy information about availability of computer code

| | |
|---|---|
| Data collection | Image capture was performed using Micromanager (https://open-imaging.com/). |
| Data analysis | Softwares used for sequencing data analysis are as follow:<br>Bowtie2 Langmead and Salzberg, 2012 http://bowtiebio.sourceforge.net/bowtie2/index.shtml<br>MACS2 https://github.com/taoliu/MACS<br>TopHat Trapnell et al., 2012 https://ccb.jhu.edu/software/tophat/index.shtml<br>Cufflinks Trapnell et al., 2012 http://cole-trapnelllab.github.io/cufflinks/<br>Deeptools2 Ramirez et al., 2016 http://deeptools.readthedocs.io/en/latest/index.html<br>SAMtools Li et al., 2009 http://samtools.sourceforge.net/<br><br>For image analysis data were analysed using Image J 2.0 and 64bit Java8.<br>Custom script for analysis of foci area can be found here: DOI: https://doi.org/10.5281/zenodo.4596974 |

For manuscripts utilizing custom algorithms or software that are central to the research but not yet described in published literature, software must be made available to editors/reviewers. We strongly encourage code deposition in a community repository (e.g. GitHub). See the Nature Research guidelines for submitting code & software for further information.

## Data

Policy information about availability of data

All manuscripts must include a data availability statement. This statement should provide the following information, where applicable:

- Accession codes, unique identifiers, or web links for publicly available datasets
- A list of figures that have associated raw data
- A description of any restrictions on data availability

All sequencing data are deposited on Geo: https://www.ncbi.nlm.nih.gov/geo/query/acc.cgi?acc=GSE130659
Custom script for analysis of foci area is deposited here: DOI: https://doi.org/10.5281/zenodo.4596974

# Field-specific reporting

Please select the one below that is the best fit for your research. If you are not sure, read the appropriate sections before making your selection.

☒ Life sciences ☐ Behavioural & social sciences ☐ Ecological, evolutionary & environmental sciences

For a reference copy of the document with all sections, see nature.com/documents/nr-reporting-summary-flat.pdf

# Life sciences study design

All studies must disclose on these points even when the disclosure is negative.

| | |
|---|---|
| Sample size | No sample size calculation was performed. For microscopy-based experiments, each experiment was always done in biological triplicates and 100 cells were counted per experiment. These are typical sample size for this type of analysis. For sequencing experiments, they were performed in two biological replicates. |
| Data exclusions | No data were excluded from the analysis |
| Replication | Experiments were performed in three biological replicates all shown in the manuscript |
| Randomization | we compared two cell lines that were either WT or mutant for BRD4 (Y430C mutation). This mutation was introduced by CRISPR-Cas9 and validation of the genotype of the cell lines was regularly tested by sequencing |
| Blinding | For microscopy experiments blinding was not possible as the differences observed between the two cell lines were too obvious. To decrease the possibility of introducing biais, the number of foci per cells were counted separately by two different persons (G.Olley and C.Boumendil) and results were then compared. For sequencing experiments the analysis is based on softwares that can't be biaised, these experiments were therefore not blinded |

# Reporting for specific materials, systems and methods

We require information from authors about some types of materials, experimental systems and methods used in many studies. Here, indicate whether each material, system or method listed is relevant to your study. If you are not sure if a list item applies to your research, read the appropriate section before selecting a response.

### Materials & experimental systems

| n/a | Involved in the study |
|---|---|
| ☐ | ☒ Antibodies |
| ☐ | ☒ Eukaryotic cell lines |
| ☐ | ☐ Palaeontology |
| ☐ | ☐ Animals and other organisms |
| ☐ | ☐ Human research participants |
| ☐ | ☐ Clinical data |

### Methods

| n/a | Involved in the study |
|---|---|
| ☐ | ☒ ChIP-seq |
| ☐ | ☒ Flow cytometry |
| ☐ | ☐ MRI-based neuroimaging |

## Antibodies

Antibodies used

BRD4 Bethyl Cat# A301-985A-M
53BP1 Novus Cat# NB100-304
Normal Rabbit IgG Santa Cruz Cat# sc-2025
CHK1 Abcam Cat# ab47574
CHK1-p Cell signaling technologies Cat# 2348
Lamin B Santa Cruz Cat# sc-374015
MAD2L2 Abcam Cat# ab180579

| | |
|---|---|
| | RIF1 A kind gift from Sara Buonomo Rabbit anti–mouse Rif1 serum 1240 42<br>RAD51 Calbiochem Cat# PC130<br>Goat anti-Rabbit IgG, secondary, Alexa Fluor 488 Invitrogen Cat# A11034<br>Donkey anti-Rabbit IgG, secondary, Alexa Fluor 586 Invitrogen Cat# A10042 |
| Validation | 53BP1, CHK1, CHK1-P, MAD2L2, laminB, RAD51 used in previous publications including publications from the Soutoglou lab, the Jackson lab, the Jacobs lab. RIF1 antibody has been validated in the Buonomo lab. DNA repair factors all showed an increased number of foci upon Neocarzinostatin treatment (see WT condition -/+NCS in this manuscript) |

## Eukaryotic cell lines

Policy information about cell lines

| | |
|---|---|
| Cell line source(s) | Y430C-BRD4 mutant and corresponding wild-type mouse embryonic stem cells (mESCs) were generated by CRISPR Cas9 genome editing in 46C mESCs as described previously (Olley et al. Nature Genetics) . NIPBL I1206del and R2298H lymphoblastoid cell lines (LCLs) were obtained from patients (Tonkin et al Nat Genetics 2004)(Gillis et al, Am. J. Hum. Genet, 2004) |
| Authentication | Validation of the Y430C mutation was performed by genomic sequencing |
| Mycoplasma contamination | Cell lines were tested negative for mycoplasma |
| Commonly misidentified lines<br>(See ICLAC register) | *Name any commonly misidentified cell lines used in the study and provide a rationale for their use.* |

## Palaeontology

| | |
|---|---|
| Specimen provenance | *Provide provenance information for specimens and describe permits that were obtained for the work (including the name of the issuing authority, the date of issue, and any identifying information).* |
| Specimen deposition | *Indicate where the specimens have been deposited to permit free access by other researchers.* |
| Dating methods | *If new dates are provided, describe how they were obtained (e.g. collection, storage, sample pretreatment and measurement), where they were obtained (i.e. lab name), the calibration program and the protocol for quality assurance OR state that no new dates are provided.* |

☐ Tick this box to confirm that the raw and calibrated dates are available in the paper or in Supplementary Information.

## Animals and other organisms

Policy information about studies involving animals; ARRIVE guidelines recommended for reporting animal research

| | |
|---|---|
| Laboratory animals | *For laboratory animals, report species, strain, sex and age OR state that the study did not involve laboratory animals.* |
| Wild animals | *Provide details on animals observed in or captured in the field; report species, sex and age where possible. Describe how animals were caught and transported and what happened to captive animals after the study (if killed, explain why and describe method; if released, say where and when) OR state that the study did not involve wild animals.* |
| Field-collected samples | *For laboratory work with field-collected samples, describe all relevant parameters such as housing, maintenance, temperature, photoperiod and end-of-experiment protocol OR state that the study did not involve samples collected from the field.* |
| Ethics oversight | *Identify the organization(s) that approved or provided guidance on the study protocol, OR state that no ethical approval or guidance was required and explain why not.* |

Note that full information on the approval of the study protocol must also be provided in the manuscript.

## Human research participants

Policy information about studies involving human research participants

| | |
|---|---|
| Population characteristics | *Describe the covariate-relevant population characteristics of the human research participants (e.g. age, gender, genotypic information, past and current diagnosis and treatment categories). If you filled out the behavioural & social sciences study design questions and have nothing to add here, write "See above."* |
| Recruitment | *Describe how participants were recruited. Outline any potential self-selection bias or other biases that may be present and how these are likely to impact results.* |
| Ethics oversight | *Identify the organization(s) that approved the study protocol.* |

Note that full information on the approval of the study protocol must also be provided in the manuscript.

# Clinical data

Policy information about clinical studies

All manuscripts should comply with the ICMJE guidelines for publication of clinical research and a completed CONSORT checklist must be included with all submissions.

| | |
|---|---|
| Clinical trial registration | *Provide the trial registration number from ClinicalTrials.gov or an equivalent agency.* |
| Study protocol | *Note where the full trial protocol can be accessed OR if not available, explain why.* |
| Data collection | *Describe the settings and locales of data collection, noting the time periods of recruitment and data collection.* |
| Outcomes | *Describe how you pre-defined primary and secondary outcome measures and how you assessed these measures.* |

# ChIP-seq

## Data deposition

- [x] Confirm that both raw and final processed data have been deposited in a public database such as GEO.
- [x] Confirm that you have deposited or provided access to graph files (e.g. BED files) for the called peaks.

| | |
|---|---|
| Data access links<br>*May remain private before publication.* | https://www.ncbi.nlm.nih.gov/geo/query/acc.cgi?acc=GSE130659 |
| Files in database submission | *Provide a list of all files available in the database submission.* |
| Genome browser session<br>(e.g. UCSC) | spike in RNA seq: https://genome.ucsc.edu/s/golley/spike%20in%203%20replicates<br>BRD4 ChIPseq https://genome.ucsc.edu/s/golley/brd4%20chip%2Dseq%202%20replicates<br>4SU seq https://genome.ucsc.edu/s/golley/4sU%202%20replicates |

## Methodology

| | |
|---|---|
| Replicates | Two biological replicates were performed |
| Sequencing depth | 50-bp single-end reads |
| Antibodies | BRD4 Bethyl Cat# A301-985A-M |
| Peak calling parameters | Peak calling was carried out using MACS2; Duplicates were filtered (filterdup, parameters:--keep-dup=1), peaks called (callpeaks, parameters: -B --nomodel -p 1e-5) and differential peaks were found (bdgdiff, parameters: -g 60 -l 250). |
| Data quality | *Describe the methods used to ensure data quality in full detail, including how many peaks are at FDR 5% and above 5-fold enrichment.* |
| Software | deepTools2 was used to make heatmaps; score files were made across specific genomic regions (computeMatrix, parameters: scale-regions scale regions –b 500 –a 500 –bs 50 –bl mm9 blacklist) and these were used to plot heatmaps (plotHeatmap, parameters: --colormap RdBluYl reverse). |

# Flow Cytometry

## Plots

Confirm that:

- [x] The axis labels state the marker and fluorochrome used (e.g. CD4-FITC).
- [x] The axis scales are clearly visible. Include numbers along axes only for bottom left plot of group (a 'group' is an analysis of identical markers).
- [x] All plots are contour plots with outliers or pseudocolor plots.
- [x] A numerical value for number of cells or percentage (with statistics) is provided.

## Methodology

| | |
|---|---|
| Sample preparation | 2 million mESCs were fixed in 70% ethanol (in PBS) at 4o 466 C for 1 hr. Fixed cells were centrifuged at 2000 rpm at 4oC for 5 min, washed twice with PBS and resuspended in 500 µl PBS. 20 µg RNase A was added and cells were incubated at 37o C for 10 min. Cells were stained with propium iodide at a final concentration of 50 µg/ml. |

| | |
|---|---|
| Instrument | Acquisition was carried out on a BD LSRFortessa cell analyser, collecting 25,000 events per sample. |
| Software | Results were analysed using BD FACSDiva 8.0.1 |
| Cell population abundance | not applicable |
| Gating strategy | gated cells were manually categorized into cell cycle stages G0/G1, S and G2/M |

☐ Tick this box to confirm that a figure exemplifying the gating strategy is provided in the Supplementary Information.

# Magnetic resonance imaging

## Experimental design

| | |
|---|---|
| Design type | *Indicate task or resting state; event-related or block design.* |
| Design specifications | *Specify the number of blocks, trials or experimental units per session and/or subject, and specify the length of each trial or block (if trials are blocked) and interval between trials.* |
| Behavioral performance measures | *State number and/or type of variables recorded (e.g. correct button press, response time) and what statistics were used to establish that the subjects were performing the task as expected (e.g. mean, range, and/or standard deviation across subjects).* |

## Acquisition

| | |
|---|---|
| Imaging type(s) | *Specify: functional, structural, diffusion, perfusion.* |
| Field strength | *Specify in Tesla* |
| Sequence & imaging parameters | *Specify the pulse sequence type (gradient echo, spin echo, etc.), imaging type (EPI, spiral, etc.), field of view, matrix size, slice thickness, orientation and TE/TR/flip angle.* |
| Area of acquisition | *State whether a whole brain scan was used OR define the area of acquisition, describing how the region was determined.* |

Diffusion MRI ☐ Used ☐ Not used

## Preprocessing

| | |
|---|---|
| Preprocessing software | *Provide detail on software version and revision number and on specific parameters (model/functions, brain extraction, segmentation, smoothing kernel size, etc.).* |
| Normalization | *If data were normalized/standardized, describe the approach(es): specify linear or non-linear and define image types used for transformation OR indicate that data were not normalized and explain rationale for lack of normalization.* |
| Normalization template | *Describe the template used for normalization/transformation, specifying subject space or group standardized space (e.g. original Talairach, MNI305, ICBM152) OR indicate that the data were not normalized.* |
| Noise and artifact removal | *Describe your procedure(s) for artifact and structured noise removal, specifying motion parameters, tissue signals and physiological signals (heart rate, respiration).* |
| Volume censoring | *Define your software and/or method and criteria for volume censoring, and state the extent of such censoring.* |

## Statistical modeling & inference

| | |
|---|---|
| Model type and settings | *Specify type (mass univariate, multivariate, RSA, predictive, etc.) and describe essential details of the model at the first and second levels (e.g. fixed, random or mixed effects; drift or auto-correlation).* |
| Effect(s) tested | *Define precise effect in terms of the task or stimulus conditions instead of psychological concepts and indicate whether ANOVA or factorial designs were used.* |

Specify type of analysis: ☐ Whole brain ☐ ROI-based ☐ Both

| | |
|---|---|
| Statistic type for inference (See Eklund et al. 2016) | *Specify voxel-wise or cluster-wise and report all relevant parameters for cluster-wise methods.* |
| Correction | *Describe the type of correction and how it is obtained for multiple comparisons (e.g. FWE, FDR, permutation or Monte Carlo).* |

## Models & analysis

| n/a | Involved in the study |
|---|---|
| ☐ | ☐ Functional and/or effective connectivity |
| ☐ | ☐ Graph analysis |
| ☐ | ☐ Multivariate modeling or predictive analysis |

| | |
|---|---|
| Functional and/or effective connectivity | *Report the measures of dependence used and the model details (e.g. Pearson correlation, partial correlation, mutual information).* |
| Graph analysis | *Report the dependent variable and connectivity measure, specifying weighted graph or binarized graph, subject- or group-level, and the global and/or node summaries used (e.g. clustering coefficient, efficiency, etc.).* |
| Multivariate modeling and predictive analysis | *Specify independent variables, features extraction and dimension reduction, model, training and evaluation metrics.* |
